# An aryloxyphenol potentiates polymyxin against multidrug-resistant *Acinetobacter baumannii*

**DOI:** 10.3389/fcimb.2026.1814325

**Published:** 2026-06-04

**Authors:** Kyungjin Lee, Yeseul Kim, Yura Lee, Yeongseo Lee, Jaesung Kwak, Seon-Yeong Kim, Jun-Seob Kim, Choong-Min Ryu, Hwi Won Seo, Dajeong Kim

**Affiliations:** 1 Infectious Disease Research Center, Korea Research Institute of Bioscience and Biotechnology, Daejeon, Republic of Korea; 2 Department of Functional Genomics, KRIBB School of Bioscience, Korea University of Science and Technology (UST), Daejeon, Republic of Korea; 3 Department of Biosystems and Bioengineering, Korea Research Institute of Bioscience and Biotechnology (KRIBB) School of Biotechnology, UST, Daejeon, Republic of Korea; 4 Infectious Diseases Therapeutic Research Center, Korea Research Institute of Chemical Technology (KRICT), Division of Medicinal Chemistry and Pharmacology, KRICT School, UST, Daejeon, Republic of Korea; 5 Department of Nano-Bioengineering, Incheon National University, Incheon, Republic of Korea; 6 Institute for New Drug Development, College of Life Science and Bioengineering, Incheon National University, Incheon, Republic of Korea

**Keywords:** antibiotic potentiation, antimicrobial resistance, aryloxyphenol, multidrug-resistant gram-negative bacteria, polymyxin adjuvant, FabI, acinetobacter baumannii, fatty acid synthesis

## Abstract

**Background:**

The increasing prevalence of antibiotic-resistant pathogens highlights the urgent need for new strategies to overcome antimicrobial resistance. Adjuvant compounds that potentiate antibiotic activity represent a promising alternative to the development of new antibiotics.

**Methods:**

Through high-throughput screening of a chemical library, an aryloxyphenol compound, PA74, was identified as a polymyxin adjuvant candidate. Checkerboard analyses, serial passage assays, cytotoxicity assays, and functional analyses using FabI-overexpressing and point-mutant strains were performed. In vivo efficacy was evaluated using a murine skin infection model.

**Results:**

PA74 potentiated polymyxin B against polymyxin-resistant Acinetobacter baumannii, Klebsiella pneumoniae, and Escherichia coli. Among six antibiotic classes tested, PA74 additionally enhanced the activity of tetracycline and chloramphenicol. Structurally, PA74 is a triclosan (5-chloro-2-(2,4-dichlorophenoxy)phenol) analogue in which the 2,4-dichlorophenol moiety is replaced by 4-amino-3-chlorophenol. PA74 exhibited a markedly lower propensity for resistance development over 15 days compared with triclosan. PA74 also showed minimal cytotoxicity across mammalian cell lines. Increased FabI abundance attenuated the synergistic effect of the polymyxin B–PA74 combination, indicating that FabI contributes to PA74-mediated activity.

**Conclusions:**

PA74 co-treatment significantly improved therapeutic outcomes in a murine skin infection model, establishing PA74 as a promising adjuvant candidate for polymyxin B against drug-resistant Gram-negative pathogens.

## 1 Introduction

Antimicrobial resistance (AMR) has become one of the most pressing global health concerns of the twenty-first century (World Health Organization, 2025). The rapid rise of resistant bacterial pathogens threatens to undermine the effectiveness of almost every class of antimicrobial agent currently in clinical use. According to the World Health Organization (WHO), bacterial AMR was directly responsible for 1.27 million deaths in 2019 and contributed to 4.95 million deaths overall (Antimicrobial Resistance, 2022; World Health Organization, 2024; 2025). Such resistance not only increases treatment failures but also jeopardizes the safety of routine medical interventions—including surgery, organ transplantation, cancer chemotherapy, and neonatal care—whose success depends heavily on effective prophylaxis and infection control (World Health Organization, 2014; Spellberg et al., 2016). The socioeconomic burden of AMR is immense, with an estimated global cost projected to reach USD 100 trillion by 2050 if current trends continue (J, 2016). The WHO, UN Environment Programme, and FAO have jointly warned that AMR represents a “silent pandemic” with the potential to reverse decades of medical progress (Programme, 2023).

In response to this escalating challenge, extensive efforts have been directed toward the development of innovative antimicrobial strategies. The development includes the discovery of novel antibiotics with previously unexplored mechanisms of action (Brown and Wright, 2016), optimization of existing agents through structural modification, and implementation of rapid diagnostic platforms that enable targeted therapy and antimicrobial stewardship (Silver, 2011; Laxminarayan et al., 2016). Parallel advances in vaccines and immunotherapies have also contributed to infection prevention. Nevertheless, the rate at which new antibiotics reach the clinic remains insufficient to offset the relentless spread of resistance (Fair and Tor, 2014). As a complementary approach, the concept of adjuvant or combination therapy, defined as the simultaneous administration of multiple antimicrobial or helper agents, has gained renewed attention (Tamma et al., 2012; Wright, 2016). By combining drugs with distinct physicochemical properties or cellular targets, such regimens can restore the activity of compromised antibiotics, minimize resistance selection, and enhance bacterial clearance (Wright, 2016; Wang et al., 2022). Accordingly, antimicrobial combination therapy is increasingly recognized as a practical and adaptive strategy to extend the useful lifespan of existing antibiotics and reinforce the therapeutic arsenal against multidrug-resistant (MDR) pathogens (Yueh and Tukey, 2016; Vaara, 2019).

Despite growing interest in membrane-targeting strategies to combat MDR pathogens, these approaches have not consistently translated into clinically safe applications. A notable example is triclosan (TCS), a broad-spectrum biocide historically incorporated into consumer products and medical disinfectants. Although TCS effectively disrupts bacterial fatty acid synthesis and membrane homeostasis, its classification as a biocide rather than a therapeutic antibiotic resulted in widespread and indiscriminate use (Russell, 2004; Halden, 2014). Concerns regarding endocrine disruption, environmental persistence, and microbiome disturbance ultimately led to regulatory restrictions by the U.S. FDA and the European Union (Halden, 2014; Weatherly and Gosse, 2017). In addition, exposure to TCS has been linked to cross-resistance to clinically important antibiotics via the upregulation of efflux pumps in certain bacterial species, further limiting its translational potential (Carey and McNamara, 2014). Interestingly, recent studies demonstrated that TCS can synergize with polymyxin B against colistin-resistant Gram-negative pathogens (Biswas et al., 2025), supporting the therapeutic relevance of fatty acid synthesis–targeting adjuvants. However, safety and resistance concerns have continued to limit the clinical applicability of TCS itself. This discrepancy between potent *in vitro* activity and acceptable clinical safety underscores the urgent need for next-generation adjuvants (Wright, 2016).

Combination treatment offers several scientific and clinical advantages that make it particularly appealing in the era of multidrug resistance. First, the concurrent use of agents with different mechanisms of action reduces the probability that a pathogen will survive via a single resistance pathway (Tamma et al., 2012). For example, β-lactams paired with β-lactamase inhibitors or colistin combined with efflux-pump inhibitors have shown the ability to circumvent established resistance mechanisms (Bush and Bradford, 2016). Secondly, combination therapy often produces a synergistic antibacterial response, in which the observed inhibition or killing exceeds the additive effect of the individual components. Such synergy can translate to reduced dosage requirements, lower toxicity, and improved clinical outcomes (Greco, 1995). Recently, polymyxin-based combination regimens have been investigated as a strategy to restore the susceptibility of multidrug-resistant Gram-negative pathogens. These studies demonstrated that co-administration of adjuvant compounds with PMB enhances outer-membrane permeability and disrupts resistance mechanisms, thereby improving antibacterial efficacy (Vaara, 2019; Kim et al., 2023). Third, combination approaches enable drug repurposing and the utilization of small molecules or adjuvants that would otherwise show minimal efficacy on their own (Wright, 2016). This reduces both the time and financial cost associated with *de-novo* antibiotic development while promoting rational use of available drugs within the One Health framework (Bank, 2017). Therefore, combination therapy represents a realistic, cost-effective, and scientifically grounded approach to mitigating the AMR crisis.

Building on this concept, we aimed to identify novel adjuvant compounds capable of enhancing the efficacy of polymyxin against resistant Gram-negative bacteria. Through a high-throughput screening, we discovered a candidate molecule designated PA74, which exhibited significant synergistic antibacterial activity when co-treated with PMB. Interestingly, the activity of PA74 was not fully recapitulated by polymyxin B nonapeptide (PMBN), a polymyxin derivative that permeabilizes the outer membrane without exerting direct bactericidal effects (Vaara, 1992). This suggests that outer membrane permeabilization alone is insufficient to account for the observed synergy and that additional bactericidal effects of intact polymyxins contribute to PA74-mediated potentiation. In addition, PA74 displayed synergistic interactions with multiple antibiotic classes, suggesting a broad adjuvant potential, while exhibiting no observable cytotoxicity. Collectively, these results highlight PA74 as a promising combination candidate for potentiating polymyxin-based and other antibacterial therapies.

## 2 Materials and methods

### 2.1 Bacterial strains, culture conditions and media

In this study, experiments were performed using clinical isolates *Acinetobacter baumannii* YCRAb357 and YCRAb667, *Klebsiella pneumoniae* SCH742, and *Pseudomonas aeruginosa* SMC-U10, which were previously reported as PMB-resistant strains (Kim et al., 2023), as well as *Escherichia coli* FORC81 (Kim et al., 2019). All bacterial strains were cultured in Luria-Bertani (LB) broth (Miller; #244620, Difco) or on LB agar plates (Cat. #24440-1201, Junsei chemical) at 37°C. Prior to each experiment, frozen stocks were streaked onto LB agar plates and incubated for 16 h at 37°C to obtain single colonies. A single colony was inoculated into 3 mL of LB broth and grown overnight at 37°C with shaking at 220 rpm. The pre-culture was then diluted 1:1000 into fresh LB medium and grown to an optical density at 600 nm (OD_600_) of 0.08–0.1, corresponding to the early exponential growth phase, for use in subsequent assays. All strains used in this study are listed in **Supplementary Table 1**.

### 2.2 High-throughput screening for polymyxin B adjuvants based on bacterial respiration

A high-throughput screening (HTS) assay was developed to identify compounds capable of potentiating polymyxin B (PMB) activity against polymyxin-resistant *A. baumannii*. *A. baumannii* YCRAb357 was precultured for 16 h at 37 °C, then diluted 1:1000 into fresh LB broth and grown to early exponential phase (OD_600_ = 0.08). Biolog Redox Dye Mix A (0.5% v/v; Cat. #74221, Biolog, CA, USA) was added to the bacterial suspension prior to the addition of PMB at a final concentration of 16 µg/mL. A total of 6,423 compounds from a chemical library were screened, with each compound dispensed at 2 µL per well into 96-well plates at a final concentration of 5 µM. Bacterial respiration was monitored using the Biolog OmniLog^®^ Phenotype MicroArray system, in which metabolic reduction of the tetrazolium dye produces a colorimetric signal proportional to respiratory activity. After 24 h of incubation at 37 °C under static conditions, absorbance was measured colorimetrically and expressed as a percentage relative to wells containing DMSO-treated control containing 16 µg/mL PMB. All experiments were performed in triplicate.

### 2.3 Analysis of bacterial growth

*A. baumannii* YCRAb667 and *P. aeruginosa* SMC-U10 were used in this study. A single colony of each strain was inoculated into 3 mL of LB medium and incubated at 37°C with shaking at 220 rpm for overnight pre-culture. The overnight cultures were then diluted 1:1000 into fresh LB medium and grown to the exponential phase (OD_600_ = 0.08–0.1) prior to subsequent experiments. PMB was prepared by two-fold serial dilution from 128 µg/mL to 0.25 µg/mL. PA70, PA72-1, PA74, and PA105 were co-administered with PMB at final concentrations of 20, 10, and 5 μM. Following treatment, bacterial cultures were incubated at 37°C for 20 h, and bacterial growth was assessed by measuring the optical density at 600 nm (OD_600_) using a microplate reader (Spark™ 10M, Tecan).

To compare the antibacterial effects of PA74 in combination with different classes of antibiotics, the PMB-resistant strain *A. baumannii* YCRAb667 was used. Bacterial cultures were prepared as described above and treated with antibiotics (4 µg/mL) in combination with PA74 (0.25 µg/mL). Aliquots of 200 μL were dispensed into each well of a 96-well plate and incubated at 37°C for 22 h. Bacterial growth was quantified by measuring OD_600_ using a Spark™ 10M microplate reader (Tecan).

### 2.4 Ethidium bromide accumulation-based efflux assay

To assess whether PA74 influences bacterial efflux activity, an ethidium bromide (EtBr) accumulation-based efflux assay was performed using *A. baumannii* YCRAb357. Overnight cultures were diluted 1:100 into fresh LB broth and grown to mid-exponential phase (OD_600_ = 0.3–0.4) at 37 °C with shaking at 210 rpm. EtBr was added to a final concentration of 1 µg/mL, and cultures were incubated under shaking conditions in the dark. Cells were harvested by centrifugation, washed twice with phosphate-buffered saline (PBS), and resuspended in an equal volume of PBS. The cell suspension was dispensed into 96-well plates at 200 µL per well, and compounds were added at the following concentrations: PMB (16 µg/mL), PA74 (2 µg/mL), PMB+PA74 combination, or CCCP (1 µg/mL) as a positive control for efflux inhibition. Fluorescence was measured every 5 min over 60 min using a Spark™ 10M microplate reader (Tecan) at excitation/emission wavelengths of 360/460 nm. Results are expressed as ΔFluorescence (Ft − F0), where F0 represents the fluorescence at time zero.

### 2.5 Membrane integrity assay

Membrane integrity was assessed using propidium iodide (PI; Invitrogen, P3566). *A. baumannii* YCRAb357 was grown from an overnight culture by 1:100 dilution into fresh LB broth to an OD_600_ of 0.5. Cells were harvested, washed once with 0.01 M PBS (pH 7.4), and resuspended in the same buffer after centrifugation at 8,000 × g for 1 min. The bacterial suspension was adjusted to OD_600_ 0.5, and PI was added to a final concentration of 0.1 μg/mL. Probe-labeled cells were dispensed into 96-well plates at 198 μL per well and allowed to stabilize for 30 min prior to compound treatment.

PA74 was added at final concentrations of 8, 4, 2, and 0 μg/mL, and CTAB (32 μg/mL) was used as a positive control. Fluorescence was measured every 5 min for a total of 50 min, beginning 10 min prior to compound addition, using a microplate reader (Spark™ 10M, Tecan) at excitation/emission wavelengths of 540/610 nm.

### 2.6 Membrane depolarization assay

Membrane depolarization was evaluated using DiSC_3_(5) (Invitrogen, D306). *A. baumannii* YCRAb357 was grown from an overnight culture by 1:100 dilution into fresh LB broth to an OD_600_ of 0.5. Cells were harvested, washed once with 5 mM HEPES buffer (pH 7.0) containing 5 mM glucose, and resuspended in the same buffer after centrifugation at 8,000 × g for 1 min. The bacterial suspension was adjusted to OD_600_ 0.5, and DiSC_3_(5) was added to a final concentration of 5 μM. Dye-labeled cells were dispensed into 96-well plates at 198 μL per well and allowed to equilibrate for 30 min prior to compound treatment.

PA74 was added at final concentrations of 8, 4, 2, and 0 μg/mL, and CTAB (32 μg/mL) was used as a positive control. Fluorescence was measured every 5 min for a total of 50 min, beginning 10 min prior to compound addition, using a microplate reader (Spark™ 10M, Tecan) at excitation/emission wavelengths of 635/670 nm.

### 2.7 Biofilm inhibition assay

Biofilm formation was assessed using a crystal violet staining method in 96-well microtiter plates. Overnight cultures of *K. pneumoniae* SCH742 were diluted 1:100 into fresh LB broth (OD_600_ ≈ 0.05) and dispensed at 300 µL per well. PA74 and PMB were added at the indicated concentrations, and plates were incubated at 37 °C for 24 h. After incubation, wells were washed twice with distilled water to remove non-adherent cells. Biofilms were stained with 0.1% (w/v) crystal violet (300 µL per well) for 20 min at room temperature, followed by two washes with distilled water. Ethanol (300 µL) was added to solubilize the stained biofilm with shaking, and absorbance was measured at OD_570_ using a Spark™ 10M microplate reader (Tecan). Results are presented as the mean ± SD of at least three independent experiments.

### 2.8 Synthesis of PA74, 2-(4-amino-3-chlorophenoxy)-5-chlorophenol

PA74 was synthesized by a slight modification of a previously reported procedure (Fujii et al., 2020). To an ice-cold dimethylformamide (DMF) solution of 4-chloro-2-methoxyphenol (266 μL, 2.2 mmol) was slowly added 1 M NaOH(aq) (1.1 equiv), followed by the addition of 2-chloro-4-fluoro-1-nitrobenzene (369 mg, 2.1 mmol) in DMF. The reaction mixture was gradually warmed to room temperature and stirred for 24 h. After completion of the reaction, H_2_O was added to the reaction mixture, and the resulting mixture was extracted with an ethyl acetate (EA). The combined organic layers were washed with brine, dried over anhydrous magnesium sulfate (MgSO_4_), filtered, and concentrated under reduced pressure. The crude residue was purified by silica gel column chromatography (EA/*n*-hexane = 1:9) to afford compound 2-chloro-4-(4-chloro-2-methoxyphenoxy)-1-nitrobenzene (627 mg, 99%): ^1^H NMR (400 MHz, CDCl_3_) δ 7.91 (d, *J* = 9.12 Hz, 1H), 7.04 – 7.01 (m, 2H), 6.97 – 6.93 (m, 2H), 6.81 (dd, *J* = 9.1, 2.72 Hz, 1H), 3.77 (s, 3H).

2-Chloro-4-(4-chloro-2-methoxyphenoxy)-1-nitrobenzene (627 mg, 2.0 mmol) was dissolved in dry CH_2_Cl_2_ (0.05 M), and the solution was cooled to 0 °C. To the stirred solution was added dropwise a 1.0 M solution of BBr_3_ (8.0 mL, 8.0 mmol) in dry CH_2_Cl_2_ at 0 °C. The reaction mixture was allowed to warm to room temperature and stirred for 4 h, then cooled again to 0 °C. To the reaction mixture was added 6% aqueous ammonia, followed by vigorous stirring at room temperature. The resulting mixture was separated and extracted with CH_2_Cl_2_. The combined organic layers were dried over anhydrous MgSO_4_ and concentrated under reduced pressure. The crude residue was purified by silica gel column chromatography (EA/*n*-hexane = 1:9 to 1:5) to afford 5-chloro-2-(3-chloro-4-nitrophenoxy)phenol (579 mg, 97%): ^1^H NMR (400 MHz, CDCl_3_) δ 7.92 (d, *J* = 9.08 Hz, 1H), 7.05 (dd, *J* = 6.6, 2.5 Hz, 2H), 6.96 – 6.90 (m, 3H), 6.45 (s, 1H).

A solution of 5-chloro-2-(3-chloro-4-nitrophenoxy)phenol (300 mg, 1.0 mmol) in EtOH (25 mL) was treated with Fe powder (76 mg, 8.0 equiv), H_2_O, and concentrated HCl (117 μL). The reaction mixture was stirred at 110 °C for 6 h. After completion of the reaction, the mixture was filtered through celite and extracted with CH_2_Cl_2_. The organic layer was washed successively with water and brine, dried over anhydrous MgSO_4_, and concentrated under reduced pressure. The crude residue was purified by silica gel column chromatography (EA/*n*-hexane = 1:3) to afford PA74 (165 mg, 61%): ^1^H NMR (400 MHz, CDCl_3_) δ 7.02 (dd, *J* = 14.36, 2.44 Hz, 2H), 6.81 – 6.74 (m, 3H), 6.69 (d, *J* = 8.64 Hz, 1H).

### 2.9 Minimal inhibitory concentration assay

MIC assays were performed following the modified guidelines of the Clinical and Laboratory Standards Institute (CLSI), document M07, 11th edition. Bacterial strains stored at −80°C were streaked onto 1.5% LB agar plates and incubated at 37°C for 16–18 h to obtain single colonies. A single colony was inoculated into 3 mL of LB broth and grown at 37°C for 16–18 h. The pre-culture was then diluted 1:1000 into fresh medium and incubated until reaching the exponential phase (OD_600_ = 0.08–0.1). The bacterial suspension was dispensed into 96-well microplates at a final volume of 200 μL per well. The highest concentration of each test compound was added to the first well and subsequently subjected to 2-fold serial dilution across the plate. Plates were incubated at 37°C for 16–18 h, and the optical density was measured using a microplate reader (Spark™ 10M, Tecan). MIC values were determined from dose–response curves as the lowest concentration achieving ≥ 90% growth inhibition relative to the untreated control.

### 2.10 Checkerboard assay

Checkerboard assay was performed to evaluate the combinatorial effects of the two compounds. Each compound was serially diluted along either the horizontal or vertical axis to generate a matrix of concentration combinations. PA74 was pre-diluted by 2-fold from its highest working concentration prior to plate loading. After incubation under the same conditions as the MIC assay, OD_600_ values were measured to assess potential interactions.

### 2.11 Antibiotics and chemical compounds

Four candidate compounds (PA70, PA72-1, PA74, and PA105) were obtained from the Korea Chemical Bank. Each compound was provided as a 5 mM stock solution dissolved in DMSO and pre-aliquoted at 30 μL per well in 96-well plates. Antibiotics used in this study were purchased from Sigma–Aldrich (St. Louis, MO, USA): polymyxin B solution (#92283), colistin (#C4461), ampicillin (#A0166), meropenem (#M2574), carbenicillin (#C1389), gentamicin (#G1264), kanamycin (#B5264), streptomycin (#S6501), chloramphenicol (#C0378), tetracycline (#T3383), ciprofloxacin (#17850), norfloxacin (#N9890), and polymyxin B nonapeptide hydrochloride (#P2076).

### 2.12 Construction of a strain *E. coli* BW25113 harboring *mcr-1* by conjugation

Bacterial conjugation was performed using *E. coli* FORC82 harboring an *mcr-1* encoding plasmid as the donor strain and *E. coli* BW25113 carrying the kanamycin-resistant vector pMo130-TelR as the recipient strain. pMo130-TelR was a gift from Kim Lee Chua (Addgene #50799). The donor strain was grown overnight in LB broth without antibiotic selection, whereas the recipient strain was grown overnight in LB broth supplemented with kanamycin (50 μg/mL). Donor and recipient cells were mixed at a 1:3 (donor:recipient) ratio and incubated at 37 °C with shaking for 4 h to allow conjugation. Following incubation, 1 mL of the mating mixture was harvested by centrifugation (5,000 × g, 4 °C, 5 min) and washed twice with 10 mM MgSO_4_. The final pellet was resuspended in 500 μL of 10 mM MgSO_4_ and spread onto LB agar plates containing colistin (4 μg/mL) and kanamycin (50 μg/mL). Plates were incubated at 37 °C for 20 h. Transconjugants were confirmed by PCR amplification of the *mcr-1* gene. The sequence of primers used in this study are listed in **Supplementary Table 2**.

### 2.13 Construction of plasmids

A plasmid for *fabI* overexpression (pCA24N-*fabI*) was obtained from a GFP-removed ASKA library available in our laboratory (Kitagawa et al., 2005). To introduce point mutation on the pCA24N-*fabI*, overlap-PCR was performed to insert G93V and F203L amino acid substitution. The sequence of primers used in this study are listed in **Supplementary Table 2**. The purified PCR products were inserted between the XhoI and NotI sites of the pCA24N plasmid. Point mutations were subsequently submitted to a commercial sequencing service for Sanger sequencing to confirm sequence integrity. Sequence data were analysed by alignment with the reference *fabI* sequence. The *fabI* overexpression plasmid and *fabI* point-mutated variants were introduced into the *E. coli* BW25113-*mcr-1* strain to allow functional comparison under polymyxin B–resistant conditions. Transformants were selected on LB agar supplemented with colistin for maintenance of the *mcr-1*-encoding plasmid and chloramphenicol for selection of the pCA24N backbone.

### 2.14 Assessment of resistance development by serial passaging

The resistance development was determined using *A. baumannii* YCRAb357. To evaluate resistance development under combination treatment conditions, PMB was maintained at a fixed concentration of 4 µg/mL throughout the experiment. Under this condition, the MICs of PA74 and TCS were determined to be 8 µg/mL and 1 µg/mL, respectively. For MIC determination, each compound was tested starting from concentrations up to 4× MIC, followed by twofold serial dilutions. For standalone resistance experiments, PA74 and TCS were tested using twofold serial dilutions, with concentration ranges designed to include 1× MIC for each compound. Bacterial growth was monitored under each condition to evaluate the emergence of resistance. After 20 h incubation at 37°C, two independent wells exhibiting OD_600_ values greater than 0.5 were sequentially pooled. The pooled culture was diluted 1:1,000 and inoculated into fresh medium for the next day’s MIC assay. This process was repeated to evaluate the potential for resistance development upon successive exposures.

### 2.15 Cytotoxicity assay

The cytotoxicity of the compounds was evaluated using the EZ-CYTOX Cell Viability Assay Kit (Dogenbio, Korea), according to the manufacturer’s instructions. Cryopreserved cells were thawed and cultured in DMEM or RPMI-1640 medium supplemented with 10% fetal bovine serum (FBS). When cells reached approximately 80–90% confluency, they were harvested using trypsin–EDTA, counted, and seeded into 96-well flat-bottom plates at a density of 2.0 × 10^4^ cells per well. Cells were allowed to stabilize for 24 h at 37°C in a humidified incubator with 5% CO_2_. Test compounds were dissolved in DMSO to prepare stock solutions and serially diluted in culture medium to final concentrations of 100, 50, 20, 10, 1, 0.1, and 0.01 μM. After stabilization, the culture medium was aspirated, and cells were treated with fresh medium containing the indicated concentrations of each compound. Cells were then incubated for 24 h at 37°C with 5% CO_2_. Following treatment, 10 μL of EZ-CYTOX reagent was added to each well, and the plates were incubated for an additional 1–2 h at 37°C. Absorbance was measured at 450 nm using a microplate reader. Blank wells contained culture medium and EZ-CYTOX reagent without cells. Cell viability was calculated as a percentage relative to the DMSO-treated control. Dose–response curves and IC50 values were determined by nonlinear regression analysis based on the Hill equation using GraphPad Prism 9.

### 2.16 Evaluation of *in vivo* antimicrobial efficacy

*In vivo* antimicrobial efficacy was evaluated using a murine skin wound infection model. Mice were anesthetized by intraperitoneal injection of 200 μL of 2.5% avertin per mouse. The dorsal hair was removed using an electric clipper followed by shaving cream application. Two symmetrical full-thickness skin wounds, each 5 mm in diameter, were generated on the dorsal surface using a biopsy punch (Kai Industries, Japan). Following wound generation, mice were randomly assigned to receive topical treatment with vehicle alone (DMSO: Tween 80:distilled water = 2:2:6, v/v/v), 80 µg/mL PMB, 20 µg/mL PA74, or a combination of 80 µg/mL PMB and 20 µg/mL PA74. For vehicle-treated controls, bacterial inoculation was performed in the same manner as in the treatment groups, and an equivalent volume of vehicle solution was applied to the wound site. Bacterial infection was established by topically applying 10 μL of a suspension of *A. baumannii* YCRAb357 at a concentration of 1.16 × 10^7^ CFU per wound to each skin wound. Twenty minutes after infection, 10 μL of the designated treatment solution was applied topically to the infected wound. The treated wounds were left undisturbed to allow absorption of both the bacterial inoculum and treatment solutions, after which mice were allowed to recover from anesthesia and monitored until full recovery. Additional treatments were administered four times within 24 h post-infection under isoflurane inhalation anesthesia. At 24 h post-infection, mice were euthanized, and infected skin tissues were excised using a biopsy punch. The excised tissues were weighed and homogenized in phosphate-buffered saline at a ratio of 1 mL per 100 mg of tissue. Tissue homogenates were serially ten-fold diluted, plated on agar plates, and incubated for bacterial enumeration. Colony-forming units were counted to determine the bacterial burden at the infection site.

### 2.17 *In vivo* toxicity test

The acute toxicity of PA74 was evaluated following a single intraperitoneal (i.p.) administration. Female C57BL/6 mice (6–7 weeks old) were randomly assigned to experimental groups (n = 3 per group). PA74 was administered at a dose of 200 mg/kg dissolved in vehicle, using the maximal soluble concentration suitable for *in vivo* administration. Control animals received an equivalent volume of vehicle alone. Following administration, mice were monitored for survival and clinical signs of toxicity for 7 days, with particular attention during the first 24 hours post-treatment. At 24 hours post-administration, blood samples were collected via retro-orbital bleeding under isoflurane anesthesia. Serum was separated by centrifugation and analyzed using a Hitachi 3100 automated biochemical analyzer (Hitachi High-Technologies Corporation, Tokyo, Japan) to assess liver and kidney function, including alanine aminotransferase (ALT), aspartate aminotransferase (AST), blood urea nitrogen (BUN), and creatinine levels. To evaluate local toxicity, intradermal (i.d.) injections were performed on the dorsal skin of mice. Each mouse received four injections at spatially separated sites: PA74 at doses of 24, 12, and 6 mg/kg, and a vehicle-alone control. This within-animal design allowed direct comparison of dose-dependent effects while minimizing inter-animal variability. The experiment was performed in triplicate (n = 3 mice). At 24 hours post-injection, mice were euthanized and skin tissues from each injection site were collected individually. Tissues were fixed in 10% neutral-buffered formalin, embedded in paraffin, and sectioned for histopathological analysis. Hematoxylin and eosin (H&E) staining was performed to evaluate dermal and epidermal integrity, inflammatory cell infiltration, and any signs of tissue damage.

### 2.18 Statistical analysis

Statistical analyses were performed using GraphPad Prism version 10 (GraphPad Software, San Diego, CA, USA). Data normality was assessed using the Shapiro–Wilk test prior to statistical analysis. Comparisons involving two independent groups were analyzed using an unpaired Student’s *t*-test. For experiments involving multiple groups and/or multiple variables, one-way or two-way analysis of variance (ANOVA) was applied as appropriate. When significant differences were detected, *post hoc* multiple comparison tests were performed to identify differences between groups, including Šídák’s test for multiple comparisons and Duncan’s multiple range test where indicated. All experiments were conducted with at least three independent biological replicates. Data are presented as mean ± SD or SEM as indicated in the Figure legends. A *P* value of < 0.05 was considered statistically significant (*P < 0.05, **P < 0.01, ***P < 0.001).

## 3 Results

### 3.1 Identification of PA74 as a PMB adjuvant against *A. baumannii*

**Figure 1:**
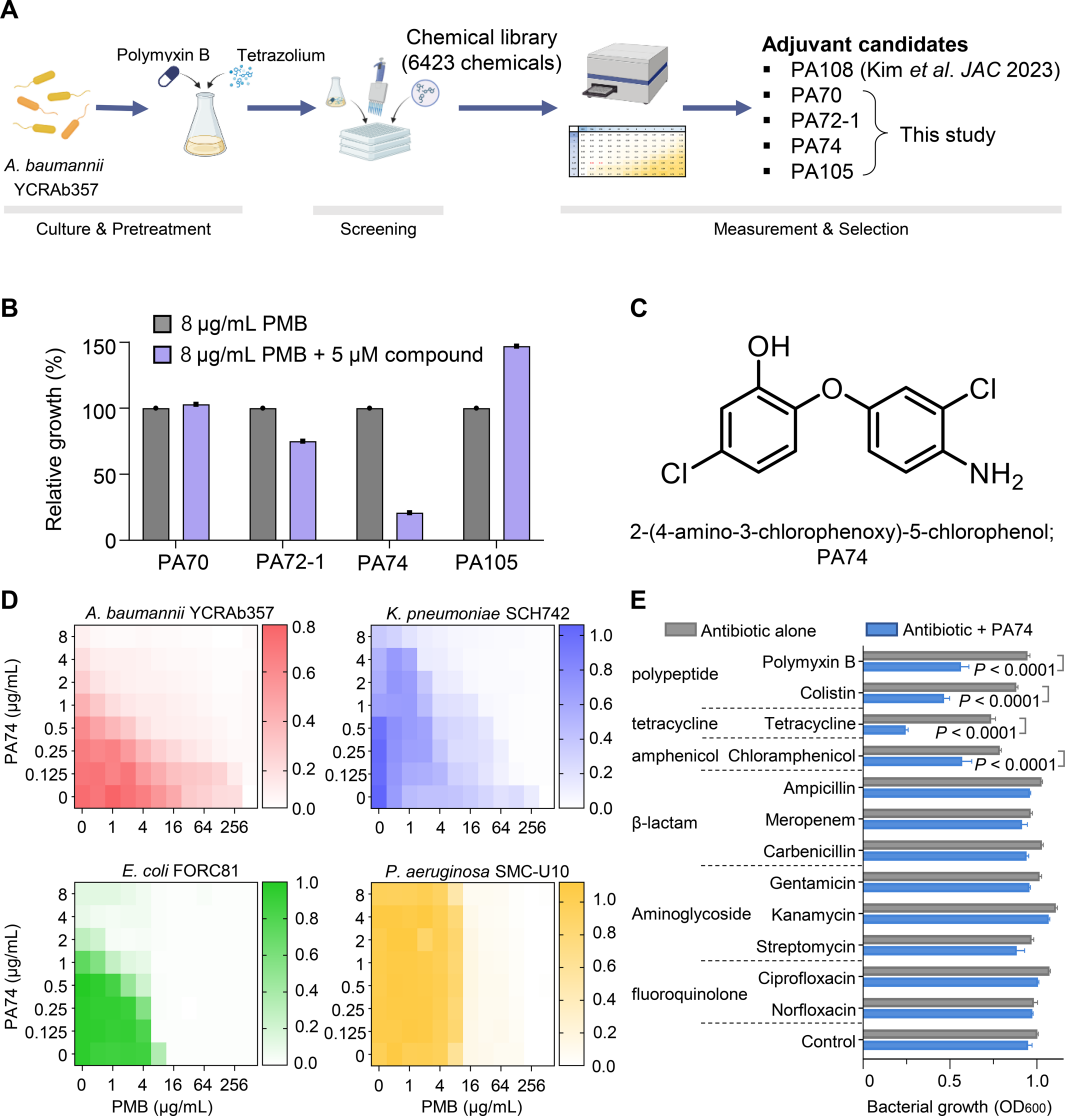
Identification of PA74 and its synergistic activity with polymyxin (B). **(A)** Schematic overview of the high-throughput phenotypic screening workflow used to identify polymyxin adjuvants. The PMB-resistant strain *A. baumannii* YCRAb357 was cultured in the presence of sub-MIC concentrations of PMB (16 µg/mL) and each compound from the chemical library (5 µM). Bacterial respiration was assessed using a tetrazolium-based redox dye system, and changes in absorbance were measured to quantify metabolic activity. **(B)** Comparative evaluation of selected candidate compounds (PA70, PA72-1, PA74, and PA105) in combination with PMB, following secondary validation using a growth-based OD_600_ assay. Each compound (5 µM) was tested with PMB (8 µg/mL) against *A. baumannii* YCRAb667. Bacterial growth was normalized to the PMB-alone treatment group (defined as 100%). Data show the mean ± SD from three independent experiments. **(C)** Chemical structure of PA74, identified as the lead adjuvant due to its superior PMB-potentiating activity. **(D)** Checkerboard assays evaluating the combined effects of PA74 and PMB against representative Gram-negative clinical isolates: *A. baumannii* YCRAb357, *K. pneumoniae* SCH742, *E. coli* FORC81, and *P. aeruginosa* SMC-U10. All strains were classified as PMB-resistant clinical isolates. Heatmaps display normalized bacterial growth (OD_600_) across concentration matrices of PA74 and PMB, with color intensity reflecting relative growth compared to the untreated control, where higher values indicate greater bacterial growth. **(E)** Evaluation of PA74 (0.25 µg/mL) in combination with antibiotics (each at 4 µg/mL) representing distinct classes and mechanisms of action against *A. baumannii* YCRAb667. Bacterial growth was measured by optical density at 600 nm after incubation under standard growth conditions.Statistical significance was determined using two-way ANOVA followed by Šídák’s multiple-comparisons test.

To identify novel small molecules capable of restoring the antibacterial efficacy of PMB against PMB-resistant *A. baumannii*, a high-throughput phenotypic screening was previously conducted using a synthetic chemical library of 6423 compounds from the Korea Chemical Bank (Kim et al., 2023). Briefly, this screening was performed using a tetrazolium-based redox dye, a redox indicator of bacterial respiration, under conditions in which PMB alone did not inhibit bacterial metabolic activity (**Figure 1A**). Among the identified candidates, PA108 was previously prioritized and reported (Kim et al., 2023). In this study, we focused on a subset of other high-ranking candidates derived from the primary screening (**Figure 1A**). The relative respiratory activity of the top-ranking candidates (PA70, PA72-1, PA74, and PA105) suppressed bacterial respiration under PMB co-treatment (**Supplementary Figure 1**). These candidates were initially classified as high-ranking based on their ability to modulate bacterial respiration in combination with PMB, rather than direct inhibition of bacterial growth. We re-evaluated four candidates in this study using a growth-based assay to validate their PMB-potentiating activity. While the initial screening was performed using *A. baumannii* YCRAb357, this secondary evaluation was conducted using *A. baumannii* YCRAb667, an independent multidrug-resistant clinical isolate (**Supplementary Table 3**), to assess the reproducibility of the observed potentiation effects across strains. This secondary validation step was designed to identify compounds with reproducible antibacterial potentiation, as respiration-based assays capture metabolic changes that do not always directly correlate with bacterial growth inhibition. To evaluate the potentiation effect under sub-inhibitory conditions, PMB was tested at concentrations corresponding to 1/16× MIC (8 µg/mL). As similar trends were observed at both concentrations, we present the data obtained at 1/16× MIC, where the intrinsic antibacterial effect of PMB is minimized, allowing clearer assessment of compound-mediated potentiation. All candidate compounds were tested at a fixed concentration of 5 μM (**Figure 1B**). Bacterial growth in the presence of PMB alone was defined as 100%, and relative growth values were calculated to enable quantitative comparison of combinatorial effects. PA74 in combination with PMB inhibited bacterial growth by 79% relative to the PMB-alone control (**Figure 1B**). In contrast, PA70 and PA105 in combination with PMB did not exhibit antibacterial activity, whereas PA72–1 in combination with PMB showed a moderate inhibitory effect, reducing bacterial growth by 25%. Based on these quantitative comparisons, PA74 was selected as the lead polymyxin adjuvant candidate for subsequent mechanistic and functional analyses (**Figure 1C**).

### 3.2 PA74 and PMB combination enhanced antibacterial activity against multiple gram-negative bacteria

To evaluate the combinatorial activity of PA74 with PMB, checkerboard assays were performed using four clinical PMB-resistant isolates representing different Gram-negative genera: *A. baumannii* YCRAb357, *K. pneumoniae* SCH742, *P. aeruginosa* SMC-U10, and *E. coli* FORC81. In *A. baumannii*, *K. pneumoniae*, and *E. coli*, heatmap analyses revealed a concentration-dependent enhancement of antibacterial activity upon co-administration of PA74 and PMB, characterized by a diagonal inhibition pattern consistent with synergistic or additive interactions. Increasing concentrations of PMB progressively reduced the concentration of PA74 required to achieve growth inhibition in these isolates (**Figure 1D**). In contrast, the *P. aeruginosa* isolate exhibited minimal or no enhancement in antibacterial activity upon combination treatment, displaying inhibition patterns comparable to those observed with single-agent exposure. This observation suggests the presence of genus-specific intrinsic factors, such as differences in membrane architecture or permeability, that may limit the activity of PA74 in *P. aeruginosa*. Similar combinatorial effects were observed when two additional clinical isolates from each genus were examined (**Supplementary Figure 2**). To further quantify these interactions, Fractional Inhibitory Concentration Index (FICI) values were calculated based on the checkerboard assay results (**Supplementary Figure 3A**). The calculated FICI values further supported synergistic to additive interactions in *A. baumannii*, *K. pneumoniae*, and *E. coli*, consistent with the observed growth inhibition patterns (**Table 1**). To further validate these combinatorial effects, bacterial growth was assessed by CFU enumeration following treatment with PA74 alone, PMB alone, or their combination. Across *A. baumannii* YCRAb357, *K. pneumoniae* SCH742, and *E. coli* FORC81, the combination treatment resulted in significant inhibition of bacterial growth compared to single treatments (**Supplementary Figure 3B**). In addition, PA74 potentiated the antibacterial activity of colistin against representative polymyxin-resistant strains, as demonstrated by checkerboard analysis (**Supplementary Figure 4**). Collectively, these results demonstrate that the PA74–PMB combination potentiates antibacterial activity against multiple clinical polymyxin-resistant *Enterobacterales* and *Acinetobacter* isolates, whereas *P. aeruginosa* remains refractory to such combinatorial enhancement.

**Table 1:** Antibacterial activity of PA74 combined with polymyxin antibiotics against Gram-negative bacteria.

Strain	Agent (µg/mL)	MIC (µg/mL)		FICI	Effect of combination
		Alone	Combi		
*A. baumannii* YCRAb357	PMB	512	16	0.063	**Synergy**
	PA74	64	2		
	Colistin	> 512	32	0.125	**Synergy**
	PA74	64	4		
	PMBN	> 512	256	0.531	Additive
	PA74	64	2		
*K. pneumoniae* SCH742	PMB	512	16	0.047	**Synergy**
	PA74	> 128	2		
	Colistin	> 512	64	0.188	**Synergy**
	PA74	> 128	8		
	PMBN	> 512	512	1.063	Indifferent
	PA74	> 128	8		
*E. coli* FORC81	PMB	16	0.5	0.063	**Synergy**
	PA74	> 128	4		
	Colistin	64	16	0.258	**Synergy**
	PA74	> 128	1		
	PMBN	> 512	512	1.004	Indifferent
	PA74	> 128	0.5		
*P. aeruginosa* SMC-U10	PMB	64	32	0.563	Additive
	PA74	> 128	8		
	Colistin	64	32	0.563	Additive
	PA74	> 128	8		
	PMBN	>512	512	1.063	Indifferent
	PA74	>128	8		

^a^MICs of PMB, colistin, PMBN, and PA74 were determined alone and in combination by checkerboard assays.

^b^FICI was calculated as follows: (MIC of PA74 in combination / MIC of PA74 alone) + (MIC of polymyxin in combination / MIC of polymyxin alone).

^c^Synergy, additive, and indifferent interactions were defined as FICI < 0.5, 0.5 ≤ FICI ≤ 1.0, and 1.0 < FICI ≤ 4.0, respectively.

^d^When the MIC exceeded the highest tested concentration, or when no complete growth inhibition was observed within the tested range, the highest tested concentration was used as a surrogate value for FICI calculation, and the resulting values should be interpreted with caution.

^e^Data were derived from **Figures 1D**, **2A**, and **Supplementary Figure S4**.

PMB, polymyxin B; PMBN, polymyxin B nonapeptide; MIC, minimum inhibitory concentration; FICI, fractional inhibitory concentration index.

Bold text indicates synergistic interactions (FICI < 0.5).

### 3.3 PA74 potentiates not only polypeptide but also tetracycline and amphenicol classes

To investigate the synergistic potential of PA74, we evaluated its effects in combination with antibiotics representing major classes with distinct mechanisms of action, including polypeptides, tetracyclines, amphenicols, β-lactams, aminoglycosides, and fluoroquinolones. This selection aimed to broadly characterize the adjuvant potential of PA74 across mechanistically diverse agents, rather than to reflect current clinical guideline recommendations. These experiments were performed using *A. baumannii* YCRAb667, because this strain allows clear evaluation of combination-specific effects, as PA74 alone does not exhibit measurable antibacterial activity under the tested conditions (0.25 µg/mL), while synergistic potentiation can be observed in combination with tested antibiotics (**Figure 1E**). It selectively and significantly potentiated the efficacy of specific antibiotic classes (**Figure 1E**). The most pronounced enhancement was observed when PA74 was combined with polymyxin-class antibiotics, including PMB and colistin (**Table 1**; **Supplementary Figure 4**). In addition, PA74 increased bacterial susceptibility to tetracycline and chloramphenicol (**Figure 1E**). In contrast, no significant changes in antibacterial activity were observed for other major classes, including fluoroquinolones (norfloxacin, ciprofloxacin), aminoglycosides (streptomycin, kanamycin, gentamicin), or β-lactams (carbenicillin, meropenem, ampicillin) (**Figure 1E**). Collectively, these findings indicate that the potentiating effect of PA74 is not limited to polypeptide class antibiotics.

### 3.4 Polymyxin B nonapeptide and PA74 combination was not fully effective

**Figure 2:**
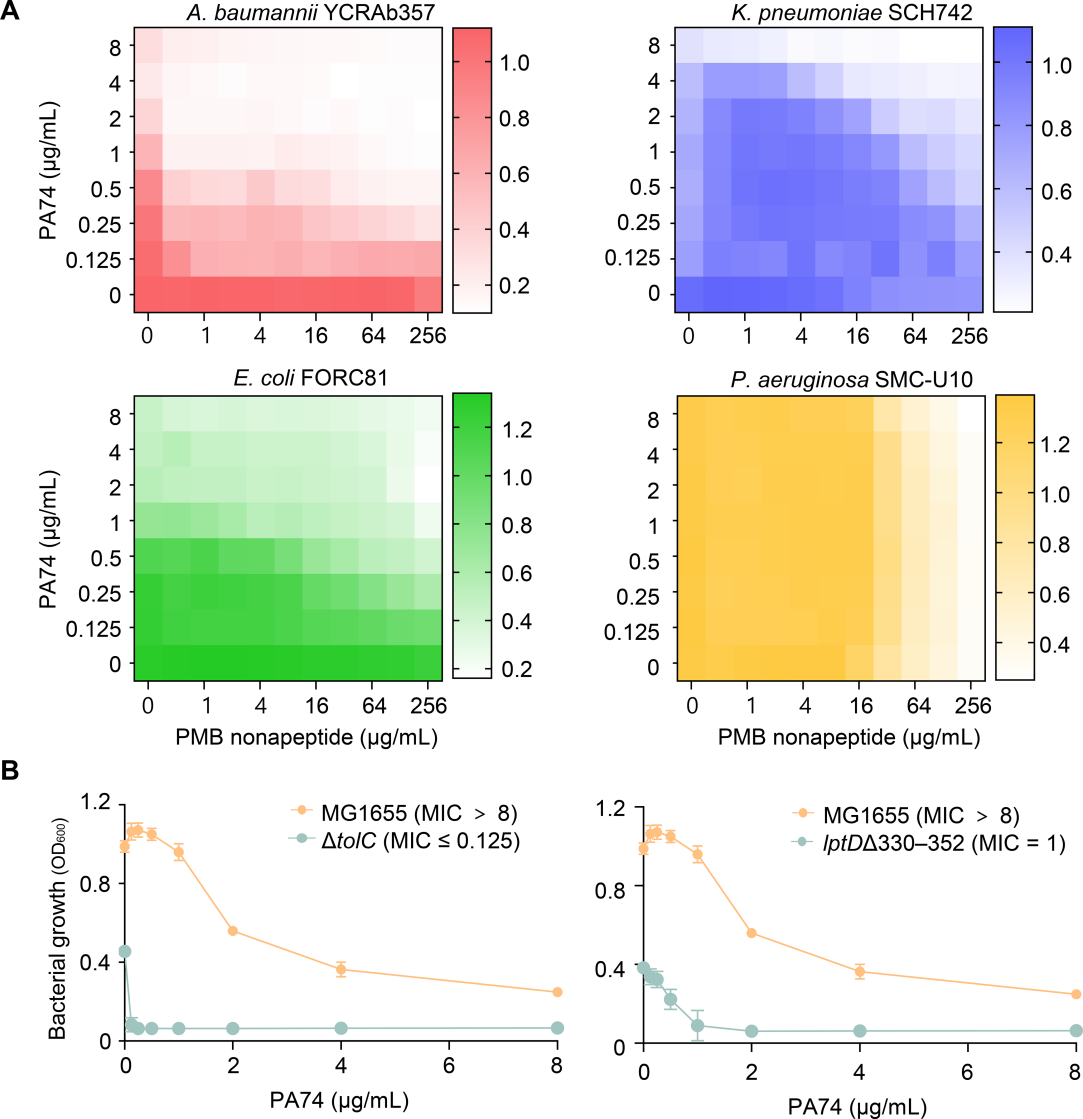
Effects of PMBN co-treatment and membrane/efflux defects on PA74 activity. **(A)** Checkerboard assays assessing the combined effects of PA74 with PMBN across four representative Gram-negative clinical isolates: *A. baumannii* YCRAb357, *K. pneumoniae* SCH742, *E. coli* FORC81, and *P. aeruginosa* SMC-U10. Heatmaps display normalized bacterial growth (OD_600_) across concentration matrices of PA74 and PMBN, with color intensity reflecting relative growth compared to the untreated control, where higher values indicate greater bacterial growth. **(B)** Dose–response analysis of PA74 in wild-type *E. coli* MG1655 and efflux- or outer membrane–defective mutants (Δ*tolC* and *lptD*Δ330–352). Bacterial growth was quantified by OD_600_ measurements across increasing concentrations of PA74 to determine relative susceptibility profiles.

Given that PA74 potentiated the activity of polymyxin-class antibiotics, we next examined whether this effect was associated with polymyxin-mediated membrane permeabilization. To address this, we evaluated the combinatorial activity of PA74 with PMBN, a polymyxin derivative that retains lipopolysaccharide-binding capacity but lacks the ability to disrupt the inner membrane (Vaara, 2019; Buchholz et al., 2024). Across three Gram-negative bacteria—*A. baumannii* YCRAb357, *K. pneumoniae* SCH742, and *E. coli* FORC81— the combination of PMBN and PA74 failed to recapitulate the enhanced antibacterial effect observed with the PMB-PA74 combination (**Figure 2A**; **Table 1**). In *P. aeruginosa* SMC-U10, no synergistic interaction was detected under any of the tested combinations (**Figure 2A**; **Table 1**). Together, these results suggest that PA74-mediated potentiation is not fully recapitulated by PMBN and that membrane-permeabilizing activity alone may be insufficient to explain the synergistic effects observed with intact polymyxins.

### 3.5 Increased intrinsic susceptibility to PA74 in efflux- and membrane-defective strains

Following the PMBN-PA74 combination experiments, which suggested that increased outer membrane (OM) permeability alone does not fully account for PA74-mediated potentiation, we next evaluated PA74 activity in strains with defined defects in drug efflux or OM integrity. Specifically, we utilized a Δ*tolC* mutant lacking the OM channel component of major multidrug efflux pumps and an *lptD*Δ330–352 mutant carrying a 23–amino acid deletion in the LPS assembly protein LptD, resulting in chronically increased OM permeability, both derived from the single-rrn-operon SQ110 background as previously described (Orelle et al., 2013). Under PA74 monotherapy conditions, both mutant strains exhibited suppressed growth and lower MIC values compared to the wild-type strain (*E. coli* MG1655). Notably, the Δ*tolC* mutant showed a markedly decreased MIC (≤ 0.125 μg/mL), reflecting a substantial increase in susceptibility. A similar trend of enhanced susceptibility was observed in the *lptD*Δ330–352 mutant. Together, these findings indicate that PA74 activity is significantly enhanced when efflux capacity is compromised or the OM barrier is weakened, supporting the notion that optimized intracellular accessibility and accumulation are critical for its antibacterial efficacy (**Figure 2B**). To further assess whether PA74 directly affects efflux activity, we performed an ethidium bromide (EtBr) accumulation assay. PA74 did not alter EtBr efflux activity compared to the untreated control, whereas CCCP, used as a positive control for efflux inhibition, showed reduced EtBr efflux, indicating that PA74 does not inhibit efflux activity (**Supplementary Figure 5**). To further determine whether PA74 directly targets the bacterial membrane, we performed outer membrane permeabilization (propidium iodide uptake) and membrane depolarization (DiSC_3_(5)) assays. PA74 alone did not induce detectable changes in membrane permeability or depolarization across the tested concentrations (**Supplementary Figure 6A** and **S6B**). These results suggest that PA74 does not primarily act by directly disrupting the bacterial membrane. Consistent with the above findings, PA74 alone did not significantly affect biofilm formation. The combination of PA74 and PMB reduced biofilm biomass in a crystal violet–based assay; however, this reduction was accompanied by a decrease in bacterial growth, indicating that the observed effect is primarily attributable to growth inhibition rather than a direct antibiofilm activity. Furthermore, no clear evidence of biofilm disruption was observed in preformed biofilms (Data not shown).

### 3.6 PA74 exhibits distinct antibacterial, cytotoxic, and resistance profiles compared to triclosan

**Figure 3:**
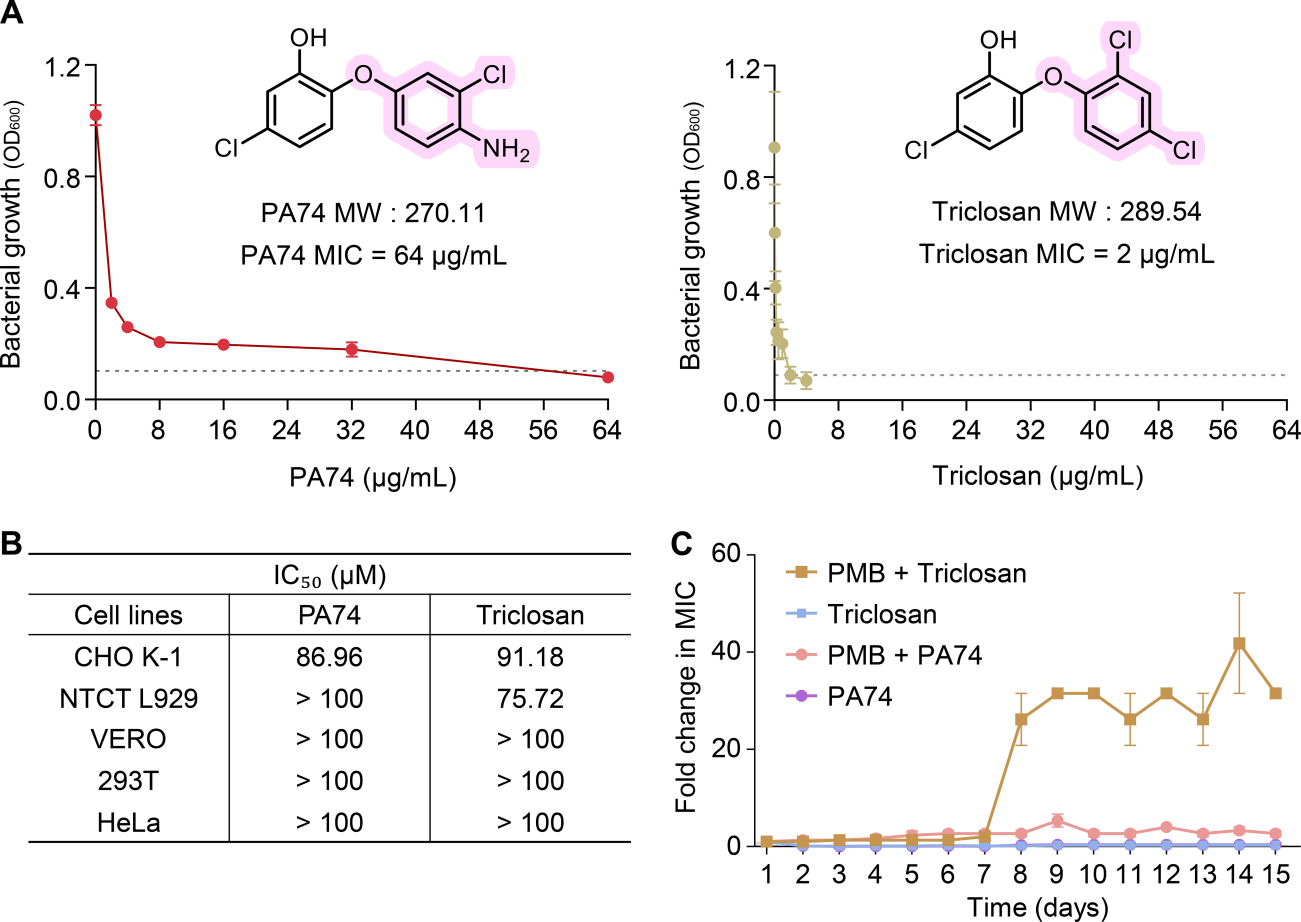
Comparison of chemical structure, intrinsic antibacterial activity, cytotoxicity, and resistance development between PA74 and triclosan. **(A)** Comparison of the minimum inhibitory concentrations (MICs) of PA74 and TCS against *A. baumannii* YCRAb357. Dose–response curves showing bacterial growth (OD_600_) across two-fold serial dilutions (128–1 μg/mL) of PA74 and TCS against *A. baumannii*. Data points represent measured OD_600_ values at each concentration and are connected by lines for visualization. A dashed line indicates the threshold corresponding to 90% growth inhibition used to define the MIC. The chemical structures of PA74 and TCS are displayed within the panels for structural comparison, with their respective molecular weights (MW) indicated below each structure. Highlighted regions (pink) indicate the shared structural scaffold and distinct substituent modifications present in PA74. MIC values were defined as the lowest concentration achieving ≥90% growth inhibition relative to the untreated control. Results represent the mean ± SD from three independent experiments. **(B)** Cytotoxicity profiles of PA74 and TCS across mammalian cell lines (CHO-K1, NCTC L929, VERO, HEK293T, and HeLa), summarized as IC_50_ values. Cell viability was assessed at concentrations up to 100 µM. **(C)** Resistance development of *A. baumannii* YCRAb357 during serial passage under selective pressure. Serial passage assays were conducted with PMB combined with either PA74 or TCS, or with PA74 and TCS alone. Under combination conditions, PMB was maintained at a fixed concentration of 4 µg/mL, and the MICs of PA74 or TCS were determined. Changes in MIC were monitored as fold changes relative to the initial MIC over a 15-day period. Results represent the mean ± SD from three independent experiments. In the graph, the purple circles (PA74) are obscured by sky blue squares (Triclosan).

Next, we examined the chemical structure of PA74 to gain insight into the basis of its potentiating activity. Structural analysis revealed that PA74 shares notable similarity with the well-characterized antibacterial TCS (**Figure 3A**). Both compounds possess a common diphenyl ether scaffold; however, a key structural difference was observed at the para position of the second aromatic ring (**Figure 3A**). PA74 was identified as a triclosan-derived analogue featuring replacement of the 2,4-dichlorophenol moiety with 4-amino-3-chlorophenol (**Figure 3A**). To assess whether the structural differences between TCS and PA74 translate into differences in intrinsic antibacterial activity, we compared their MICs against *A. baumannii*. TCS exhibited an MIC of 2 µg/mL against *A. baumannii* YCRAb357, whereas PA74 displayed a markedly higher MIC of 64 µg/mL, indicating reduced antibacterial activity relative to TCS (**Figure 3A**). When used in combination with PMB, TCS exhibited antibacterial activity at lower concentrations than PA74 in *A. baumannii*, *K. pneumoniae*, and *E. coli* (**Supplementary Figure 7**). To evaluate the cytotoxicity of PA74, its half-maximal inhibitory concentration (IC_50_) was determined across five mammalian cell lines and compared with that of TCS (**Figure 3B**). Cells were treated with varying concentrations (0.01–100 μM) of each compound for 24 h, and cell viability was determined relative to untreated controls. PA74 showed minimal cytotoxicity, with IC_50_ values ≥ 86.96 µM (95% CI: 79.02–95.53 μM) (**Figure 3B**). Notably, PA74 displayed comparable or lower cytotoxicity than TCS, particularly in fibroblast-derived NCTC L929 cells, where TCS exhibited an IC_50_ of 75.72 µM (95% CI: 69.00–82.91 μM) (**Figure 3B**).

We next determined whether PA74 differed from TCS in its propensity to induce resistance during prolonged exposure when used in combination with PMB. The fold change in MIC represents the relative increase in resistance compared to the initial MIC over serial passages. During repeated exposure to TCS plus PMB, a rapid and progressive increase in MIC was observed, beginning around day 8 and reaching approximately a 40-fold increase relative to the initial MIC by day 15 (**Figure 3C**). In contrast, no measurable increase in MIC was observed for PA74 during serial passaging, and the MIC of PA74 remained stable throughout the 15-day experimental period both as a standalone treatment and in combination with PMB (**Figure 3C**). Likewise, TCS did not exhibit significant resistance development under standalone treatment conditions. These results indicate that, although TCS alone did not readily induce resistance, its combination with PMB imposes a substantial resistance burden, whereas the PA74–PMB combination did not promote resistance development under the tested conditions. Collectively, these results indicate that PA74 exhibits a more stable resistance profile relative to TCS despite its reduced intrinsic antibacterial activity, supporting its further development as a polymyxin adjuvant distinct from TCS.

### 3.7 Mechanistic implication of FabI in PA74-mediated polymyxin B potentiation

**Figure 4:**
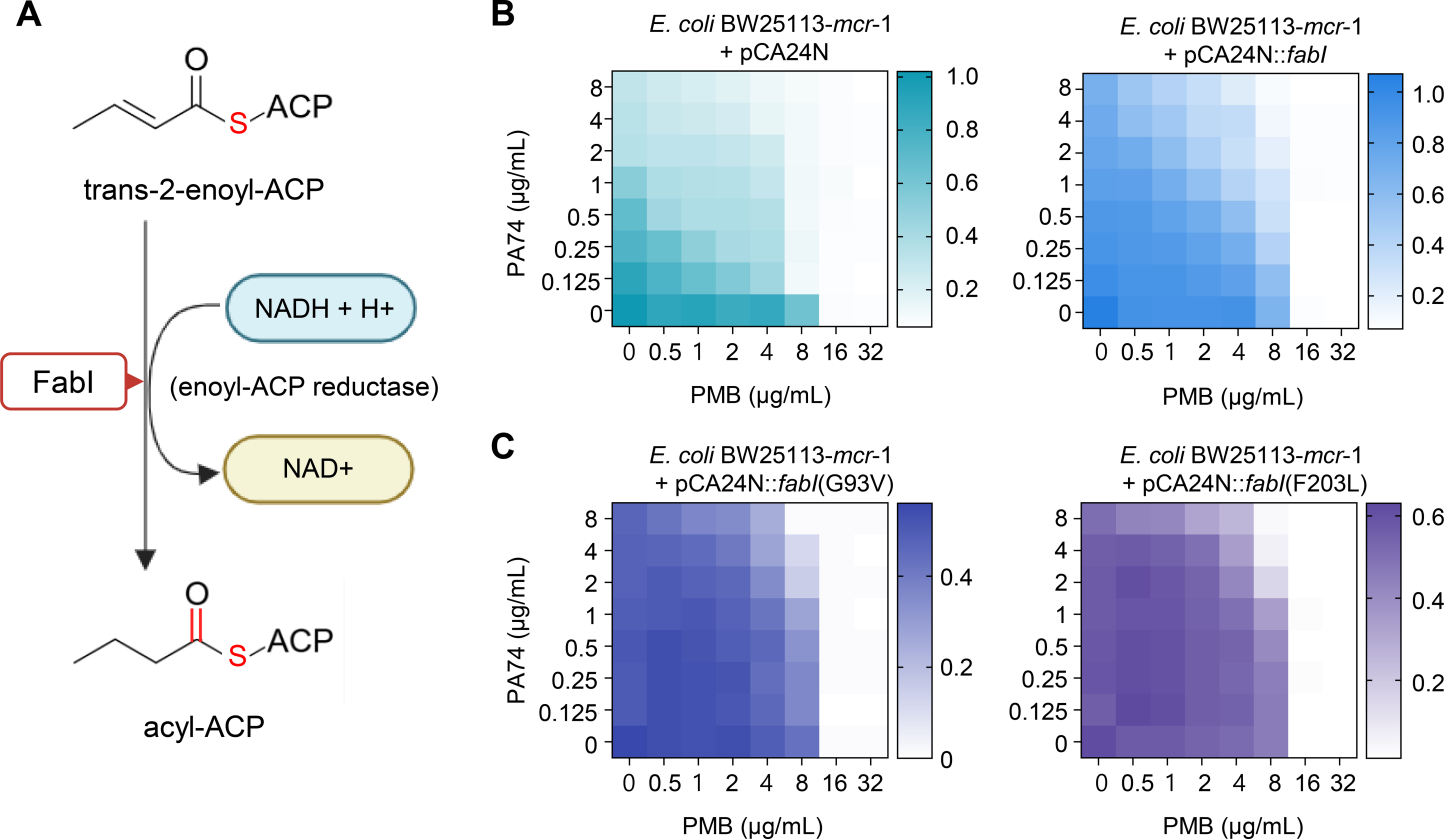
Influence of FabI abundance and mutation on PA74-mediated potentiation. **(A)** Schematic illustration of a key step in the bacterial fatty acid biosynthesis (FAS II) pathway, highlighting the role of FabI as an enoyl–acyl carrier protein (ACP) reductase that catalyzes the NADH-dependent reduction of trans-2-enoyl-ACP to acyl-ACP. **(B)** Checkerboard assays evaluating the effect of FabI abundance on the potentiating activity of PA74 in combination with PMB. The measured OD_600_ values were visualized as heatmaps, where color intensity corresponds to relative bacterial growth levels. The left panel shows results obtained with *E. coli* BW25113-*mcr-1* harboring the empty vector pCA24N (p), whereas the right panel shows results from BW25113-*mcr-1* carrying pCA24N::*fabI* (p::*fabI*). FabI overexpression was induced with 50 μM IPTG. The data show representative results from three independent experiments. **(C)** Checkerboard assays showing the combinatorial effects of PA74 and PMB in BW25113-*mcr-1* harboring pCA24N::*fabI*(G93V) (left) or pCA24N::*fabI*(F203L) (right). The measured OD_600_ values were visualized as heatmaps, where color intensity corresponds to relative bacterial growth levels. The data show representative results from three independent experiments.

TCS is a broad-spectrum antimicrobial agent reported to affect multiple cellular targets, including membrane function and macromolecular synthesis, particularly at high concentrations (Heath et al., 1998; Russell, 2004). Among these targets, enoyl–acyl carrier protein reductase (FabI) is the most well-characterized and physiologically relevant, serving as a terminal and rate-limiting enzyme in the bacterial type II fatty acid biosynthesis (FAS II) pathway (McMurry et al., 1998). FabI catalyzes the NADH-dependent reduction of trans-2-enoyl-ACP to acyl-ACP, a critical step required for fatty acid elongation and membrane lipid homeostasis (**Figure 4A**). Inhibition of FabI disrupts *de novo* fatty acid synthesis, leading to compromised membrane integrity and bacterial growth inhibition (Heath et al., 1999). Given the structural similarity between PA74 and TCS, we hypothesized that FabI may contribute to the adjuvant activity of PA74. To evaluate this hypothesis, checkerboard assays were performed using the BW25113-*mcr-1* strain harboring pCA24N-*fabI* construct. Overexpression of the *fabI* gene reduced the potentiating effect of PA74, as higher concentrations of PA74 were required to achieve synergy with PMB compared with the empty vector control (**Figure 4B**). Given that Gly93 and Phe203 are critical residues within the triclosan-binding pocket of FabI and that substitutions at these positions are known to confer TCS resistance by disrupting inhibitor binding (Sivaraman et al., 2003), we introduced the corresponding G93V and F203L mutations into FabI to evaluate their impact on PA74-PMB synergy. In these mutant strains, the concentration of PA74 required to restore PMB activity was increased beyond that observed in the wild-type FabI overexpression strain (**Figure 4C**). Although PA74 exhibited reduced intrinsic antibacterial activity compared with TCS, the attenuation of PA74-PMB synergy observed in strains harboring the G93V and F203L FabI mutations suggests that PA74 engages a similar binding pocket within FabI. Given that these residues are critical for TCS interaction and resistance, the diminished potentiating effect of PA74 in the corresponding mutant strains supports that PA74 and TCS may share overlapping structural determinants for FabI binding.

### 3.8 Antimicrobial efficacy of PA74 in a murine skin wound infection model

**Figure 5:**
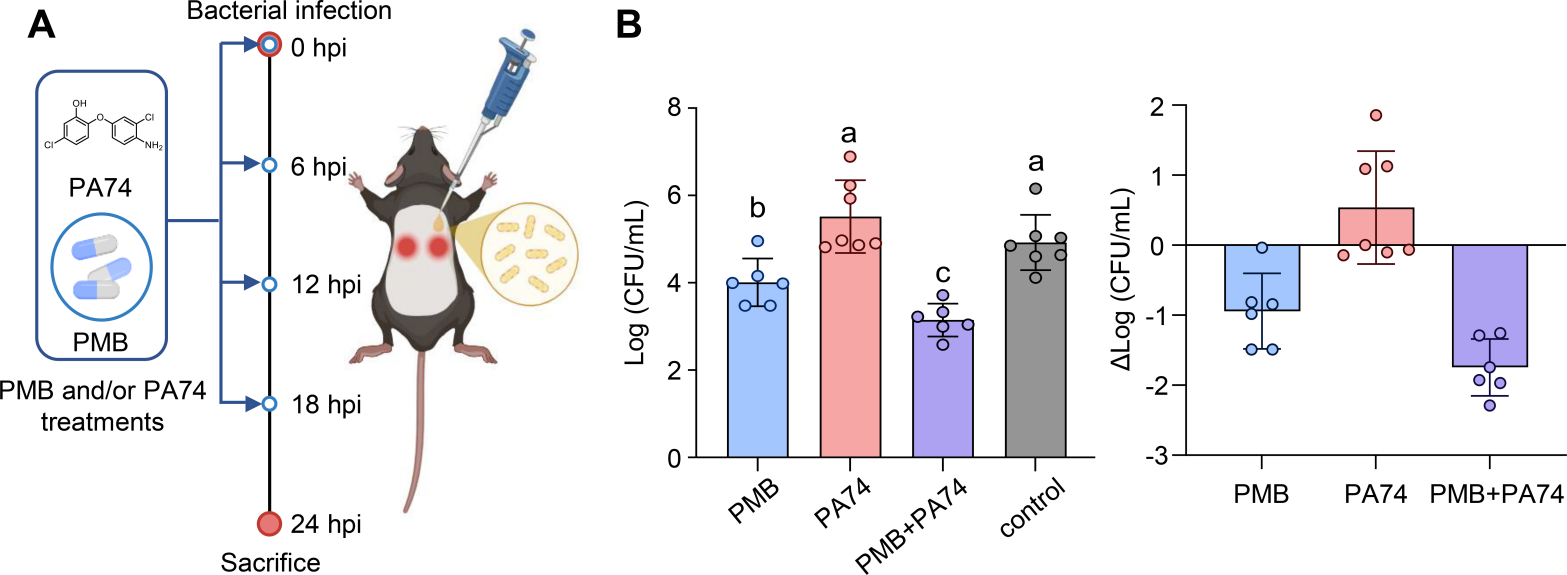
*In vivo* efficacy of the PA74–polymyxin B combination in a murine skin infection model. **(A)** Schematic overview of the experimental design for the *A. baumannii* skin infection model. Mice were topically inoculated with *A. baumannii* YCRAb357 (1.16 × 10^7^ CFU per wound). Twenty minutes after infection, vehicle control, PA74 alone, PMB alone, or a combination of PA74 and PMB was applied topically to the wound site. Treatments were administered four times within 24 h post-infection. At 24 h post-infection, infected skin tissues were excised, homogenized, serially diluted, and plated for colony-forming unit (CFU) enumeration to assess bacterial burden at the infection site. **(B)** Bacterial loads recovered from infected skin tissues (left) and the corresponding log reduction (right) following treatment. Bacterial counts are expressed as log_10_(CFU/mL). Statistical significance was determined by one-way ANOVA followed by Duncan’s multiple range test; different letters above bars indicate statistically significant differences (*P* < 0.05).

After confirming that PA74 targets FabI and exhibits a favorable cytotoxicity and resistance profile, we evaluated *in vivo* antimicrobial efficacy of PA74 using a murine skin wound infection model (**Figure 5A**). Mice were topically inoculated with *A. baumannii* YCRAb357 (1.16 × 10^7^ CFU per wound). Twenty minutes after infection, vehicle control, PA74 alone, PMB alone, or a combination of PA74 and PMB was applied topically to the wound site according to the experimental scheme illustrated in **Figure 5A**. At 24 h post-infection, treatment with PA74 alone resulted in an approximately 0.6 log CFU/mL increase in bacterial burden compared to the vehicle control, whereas PMB alone led to a reduction of approximately 0.9 log CFU/mL (**Figure 5B**). In contrast, combination treatment with PMB and PA74 produced a pronounced reduction of approximately 2 log CFU/mL relative to the control group, representing antibacterial effect among all treatment groups (**Figure 5B**). Collectively, these results demonstrate that PA74 enhances the *in vivo* antibacterial activity of PMB, supporting its role as an effective antimicrobial adjuvant.

### 3.9 PA74 exhibits no detectable acute systemic or local toxicity *in vivo*

To evaluate the *in vivo* toxicity of PA74, both acute systemic toxicity and local skin toxicity were assessed following a single-dose administration. All mice receiving PA74 at 200 mg/kg via intraperitoneal injection survived throughout the 7-day observation period without any observable clinical signs of toxicity or body weight loss (data not shown). Serum biochemical analysis revealed no significant alterations in liver or kidney function markers, including blood urea nitrogen (BUN), creatinine (CRE), aspartate aminotransferase (AST), and alanine aminotransferase (ALT), compared to vehicle-treated controls (**Supplementary Figure 8A**).

To further assess local toxicity, intradermal administration of PA74 was performed at multiple concentrations within the same animal. Histopathological examination of skin tissues collected from all injection sites showed no evidence of inflammatory cell infiltration, epidermal or dermal structural disruption, or other treatment-related lesions across all tested concentrations (**Supplementary Figure 8B**).

## 4 Discussion

Our study demonstrates that PA74 functions as an adjuvant that enhances the antibacterial activity of PMB. While PA74 alone exhibited minimal intrinsic antibacterial activity, its combination with PMB potentiated antibacterial effects against *A. baumannii*, *K. pneumoniae*, and *E. coli*. In contrast, no enhancement was observed in *P. aeruginosa*, indicating that the adjuvant activity of PA74 is genus-specific rather than universally applicable across Gram-negative bacteria. In *A. baumannii*, PA74 further broadened its potentiating activity beyond polymyxins to include chloramphenicol and tetracycline, suggesting adjuvant activity across multiple antibiotic classes. Together with structural characterization and FabI-related functional analyses, these results provide mechanistic clues regarding PA74 activity. In addition, analyses of resistance development and animal infection models support the applicability of PA74 as a polymyxin adjuvant.

The adjuvant effect of PA74 on three classes of antibiotics indicates that its combination activity does not arise from nonspecific potentiation, but instead reflects selective interactions with antibiotic targets and cell envelope–associated processes. In *A. baumannii*, PA74 enhanced antibacterial activity when combined with polymyxins (**Figure 1D**), and this effect was also observed with colistin, another polymyxin-class antibiotic (**Supplementary Figure 4**), as well as selectively with chloramphenicol and tetracycline (**Figure 1E**). Notably, these antibiotics share a common intracellular target—the bacterial ribosome—and are strongly influenced by efflux-mediated resistance, a dominant mechanism in Gram-negative bacteria (Nikaido, 1996; Li et al., 2015; Du et al., 2018). This convergence suggests that PA74 activity is preferentially manifested when intracellular drug accumulation is limiting or when envelope-associated stress responses modulate susceptibility, rather than through a generalized increase in membrane permeability. This lack of synergy observed in *P. aeruginosa* across all tested combinations (**Figure 1D**; **Supplementary Figure 2**) may be attributed to species-specific intrinsic resistance mechanisms. *P. aeruginosa* possesses exceptionally low outer membrane permeability, which inherently limits the intracellular accumulation of hydrophobic compounds such as PA74 (Hancock and Brinkman, 2002). In addition, *P. aeruginosa* encodes multiple highly active RND-type efflux pump systems, including MexAB-OprM, MexCD-OprJ, and MexXY-OprM, which collectively mediate broad-spectrum efflux of structurally diverse compounds and represent a major determinant of intrinsic multidrug resistance (Poole, 2004; Li et al., 2015). Furthermore, *P. aeruginosa* employs robust polymyxin resistance mechanisms, including lipid A modification mediated by the PhoPQ and PmrAB two-component regulatory systems, which reduce the net negative charge of the outer membrane and thereby diminish the ability of polymyxin B to permeabilize the membrane and facilitate PA74 entry (Needham and Trent, 2013). Together, these features may limit PA74 accumulation and reduce the effectiveness of polymyxin-mediated membrane perturbation, thereby attenuating combinatorial activity in *P. aeruginosa.*

The mechanistic specificity of PA74 is further elucidated by comparative experiments with PMBN that abolish antibiotic activity but retain membrane-permeabilizing capacity (**Figure 2A**). PMB disrupts both the outer and inner membranes, leading to depolarization and collapse of energy homeostasis (Storm et al., 1977; Velkov et al., 2010). In contrast, PMBN selectively permeabilizes the outer membrane without causing inner membrane disruption or direct bactericidal effects (Vaara and Vaara, 1983; Vaara, 1992). In this study, the combination of PA74 with PMBN exhibited weaker synergy than the PA74–PMB combination (**Figure 2A**). In contrast to the pronounced contour shifts observed with intact PMB (**Figure 1D**), PMBN co-treatment failed to reproduce comparable enhancement. These findings indicate that membrane permeabilization alone is insufficient to fully account for the observed potentiation and that the bactericidal activity of intact PMB contributes to the synergistic interaction. Notably, the MIC of PA74 was markedly reduced in an outer membrane–defective *lptD* mutant compared with the wild-type strain (**Figure 2B**), suggesting that the outer membrane acts as a permeability barrier limiting PA74 entry. Consistently, disruption of major efflux systems also significantly decreased the MIC of PA74 (**Figure 2B**), indicating that intracellular accumulation of PA74 is influenced by active efflux. Taken together, these findings support a model in which PA74 requires intracellular access for antibacterial activity and is subject to both permeability and efflux barriers. This intracellular mode of action is consistent with FabI being a potential functional target of PA74.

Given that PA74 is structurally derived from TCS, a well-characterized inhibitor of the enoyl–acyl carrier protein reductase FabI, perturbation of fatty acid biosynthesis and membrane lipid homeostasis represents a plausible mechanistic axis underlying the observed synergy (Heath et al., 1998; McMurry et al., 1998). Consistent with this model, modulation of *fabI* expression and substitutions at key residues within the FabI binding pocket caused concentration-dependent shifts in PA74-mediated potentiation (**Figure 4B, C**). These findings support FabI as a key functional determinant of PA74 activity, indicating that increased FabI abundance may titrate intracellular PA74 availability, while point mutations likely weaken target–ligand interactions, thereby shifting the concentration dependence of PA74-mediated effects rather than abolishing them entirely. Although FabI mutations attenuated PA74 potentiation, the synergistic effect was not completely abolished (**Figure 4C**). This observation can be explained by at least two non-mutually exclusive mechanisms. First, as a triclosan-derived compound, PA74 may retain the capacity to exert additional, FabI-independent effects at the elevated concentrations. Previous studies demonstrated that triclosan-like molecules can interact directly with membrane-associated proteins and the lipid bilayer itself at higher concentrations, thereby imposing multilayered envelope stress beyond FabI inhibition alone (Russell, 2004; Weatherly and Gosse, 2017). Secondly, our results are consistent with the formation of a “lethal bottleneck,” in which partial preservation of FabI activity remains insufficient to meet the heightened demand for membrane lipid synthesis imposed by PMB-induced membrane damage. Under such conditions, even residual fatty acid biosynthetic capacity can be overwhelmed, pushing the cell beyond a threshold compatible with envelope integrity and resulting in irreversible membrane failure. In this framework, PA74 does not function as a direct bactericidal agent but contributes to a static, non-lytic envelope stress state, at least in part by constraining membrane lipid supply through FabI inhibition. This enforced vulnerability becomes lethal only when superimposed on the dynamic membrane disruption caused by PMB (**Figures 1D**, **2B**). In other words, PA74 establishes a latent structural defect that is unmasked and amplified by PMB-mediated envelope damage. This interpretation is further supported by the PMBN comparison experiments, in which outer membrane permeabilization in the absence of inner membrane disruption or energetic collapse was insufficient to convert PA74-induced lipid limitation into bactericidal synergy (**Figure 2A**). This framework provides a mechanistic explanation for the selective and context-dependent potentiation mediated by PA74, and distinguishes it from TCS, whose antibacterial activity is more directly attributable to FabI inhibition alone (Heath et al., 1998; McMurry et al., 1998).

PA74 reduced its intrinsic antibacterial activity compared to TCS and showed a generally comparable or modestly improved tolerability profile in selected mammalian cell lines, although this effect was not consistent across all tested cell types (**Figure 3B**). Accordingly, PA74 is best characterized as an adjuvant optimized to selectively enhance the efficacy of existing antibiotics while preserving host cell safety. This profile distinguishes PA74 from conventional triclosan-class compounds by addressing their long-standing limitations and aligns more closely with the clinical requirements of combination therapy. This balance between antibacterial efficacy and host safety is particularly significant when PA74 is applied as an adjuvant to PMB. Previous studies demonstrated that TCS can synergize with PMB to overcome resistance in Gram-negative pathogens, highlighting the therapeutic potential of targeting fatty acid synthesis in combination regimens (Biswas et al., 2025). However, the clinical applicability of TCS has remained constrained by concerns regarding cytotoxicity and the potential for selecting cross-resistance. In contrast, PA74 features structural modification of the TCS scaffold that reduces non-specific toxicity while preserving synergistic activity. By addressing key translational barriers associated with conventional triclosan-class compounds, PA74 represents a safer and more refined approach to antimicrobial potentiation. This improved safety profile, characterized by reduced host cell toxicity, provided a robust rationale for evaluating the therapeutic potential of the PA74–PMB combination *in vivo*. Consistent with this rationale, PA74 was well tolerated in *in vivo* toxicity assessments with no observable adverse effects under the tested conditions (**Supplementary Figure 8**), supporting its safety profile beyond *in vitro* assays. Furthermore, when evaluated in an infection model, the PA74–PMB regimen maintained significant antibacterial efficacy in a skin infection setting, corroborating its favorable *in vitro* safety profile (**Figure 5**). Taken together, the PA74–PMB combination offers a viable strategy to extend the clinical utility of polymyxin antibiotics, whose systemic use has historically been limited by toxicity. Polymyxin B has been reported to be used in topical formulations for localized infections, and the present findings suggest that incorporation of a mechanism-specific adjuvant can further augment therapeutic outcomes in such settings (Zavascki et al., 2007). Consequently, PA74 represents a promising candidate from a drug-repurposing perspective, with marked clinical potential as part of a combination antibacterial strategy for the treatment of localized infections.

While our study provides strong functional and phenotypic evidence supporting PA74 as a mechanism-specific adjuvant to polymyxin B, several limitations should be acknowledged. First, although FabI emerged as a key functional determinant, the synergy observed at higher concentrations suggests the potential involvement of additional envelope-associated stress pathways. Future investigations incorporating transcriptomic and lipidomic analyses will be necessary to delineate how PA74 influences membrane permeability and associated regulatory networks at a systems level. Secondly, therapeutic efficacy was evaluated primarily in a localized skin infection model. Consequently, key pharmacological parameters—including tissue penetration, local retention, and PK/PD relationships—remain to be fully elucidated. Given the well-documented systemic toxicity of PMB, determining whether PA74 enables a clinically meaningful dose-sparing effect in systemic infection models represents an important direction for future studies. Finally, the long-term impact of repeated exposure on skin microbiome stability remains an open and clinically relevant question.

In conclusion, PA74 is a structurally refined TCS analogue that mitigates the cytotoxic limitations of conventional triclosan-class molecules while enabling potent antibacterial synergy with polymyxin B. By selectively constraining fatty acid biosynthesis and exploiting polymyxin-induced envelope stress, PA74 establishes a context-dependent vulnerability that enhances antibacterial efficacy in both *in vitro* and *in vivo* settings. Importantly, these findings provide mechanistic insight into how modulation of envelope integrity, efflux constraints, and intracellular target accessibility can be strategically leveraged to overcome intrinsic resistance in Gram-negative bacteria. Rather than functioning solely as a standalone bactericidal agent, PA74 exemplifies a mechanism-guided adjuvant approach that may help restore the activity of existing antibiotics. In the broader context of multidrug-resistant Gram-negative bacteria, such targeted combination strategies represent a rational framework for improving therapeutic efficacy while potentially limiting resistance development.

## Data Availability

The original contributions presented in the study are included in the article/**Supplementary Material**. Further inquiries can be directed to the corresponding authors.
