# Supplementary material for: An aryloxyphenol potentiates polymyxin against multidrug-resistant Acinetobacter baumannii
Source: Front Cell Infect Microbiol. 2026 Jun 4;16:1814325. doi: 10.3389/fcimb.2026.1814325 (PMC13275670; doi:10.3389/fcimb.2026.1814325)
Supplement: Supplementary file 1 [file DataSheet1.docx]

Supplementary Material

# Supplementary Figures


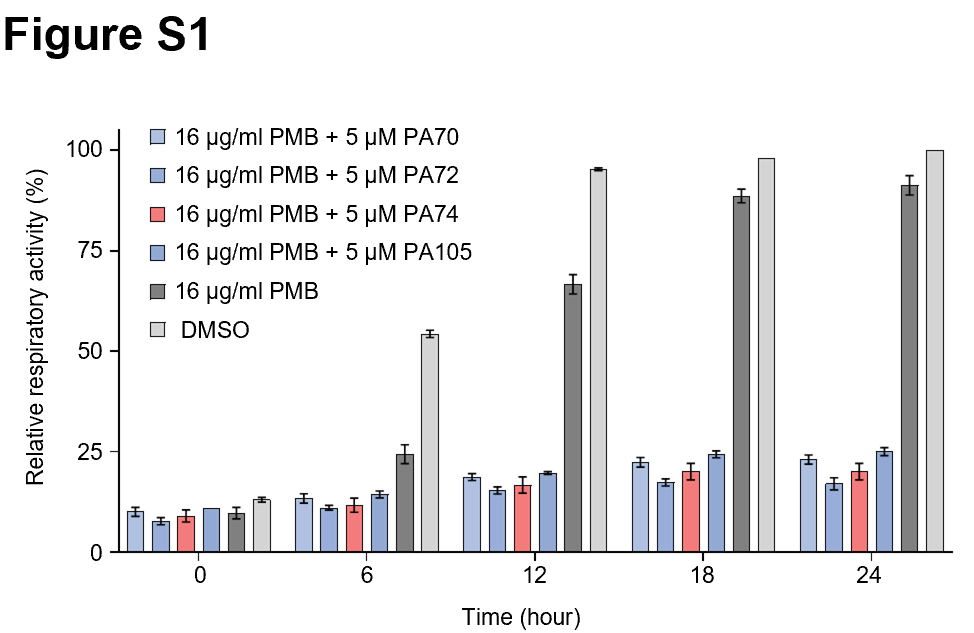


**Supplementary Figure S1.** High-throughput screening of polymyxin B adjuvant candidates based on bacterial respiration. Relative respiratory activity of *Acinetobacter baumannii* YCRAb357 was measured over 24 h in the presence of polymyxin B (PMB) (16 µg/mL) combined with each of the four candidate compounds (PA70, PA72-1, PA74, and PA105) at 5 µM, using the Biolog OmniLog® Phenotype MicroArray system. Bacterial respiration was assessed by colorimetric reduction of tetrazolium dye and expressed as a percentage relative to the DMSO-treated control. Data represent the mean ± SD from three independent experiments.


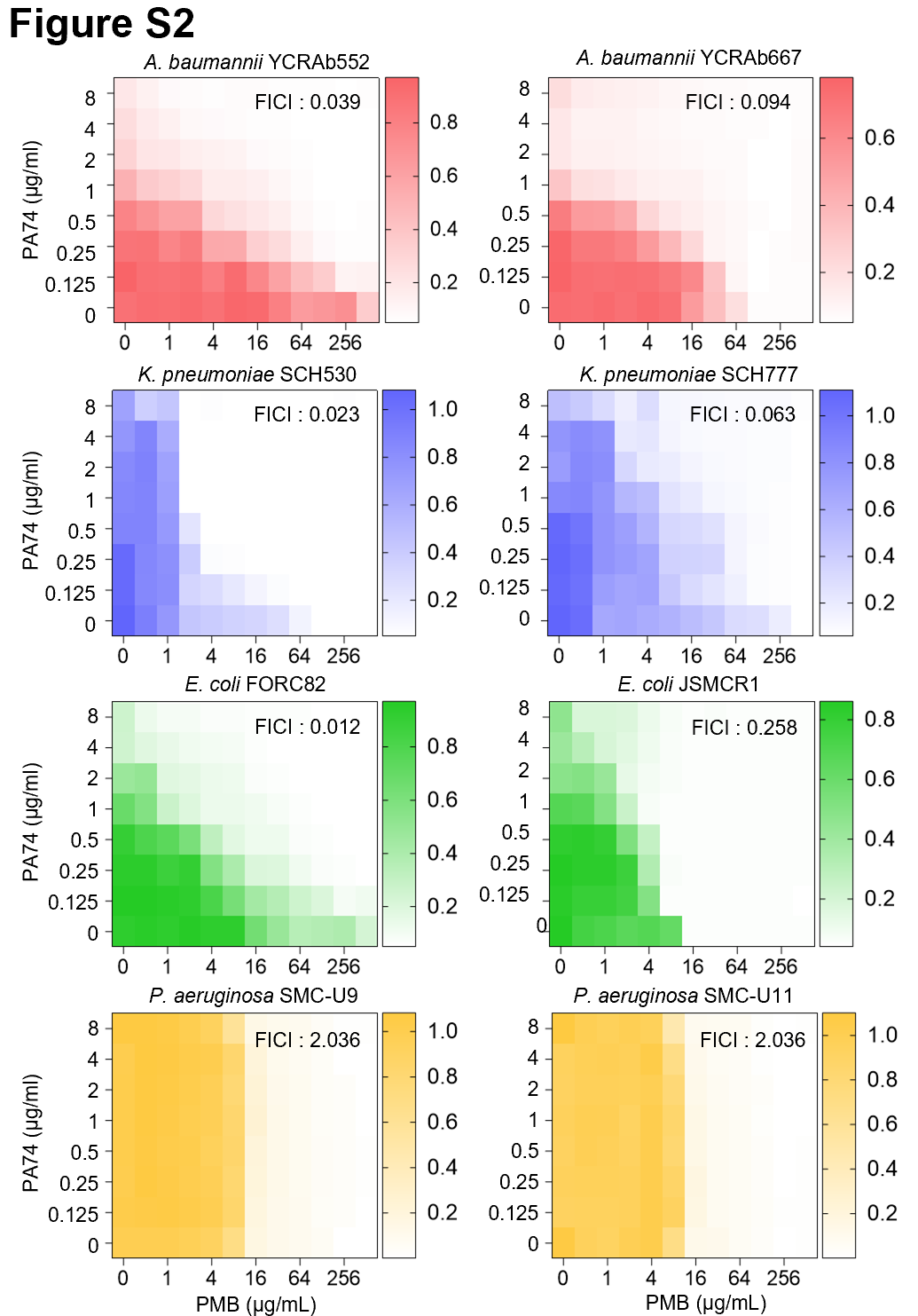


**Supplementary Figure S2.** Checkerboard analysis of the PMB–PA74 combination across multiple clinical strains. Checkerboard assays evaluating the combined effects of PA74 and PMB against representative Gram-negative bacterial strains. Normalized bacterial growth (OD_600_) is shown across concentration matrices of PA74 and PMB, with color intensity reflecting relative growth compared to the untreated control, where higher values indicate greater bacterial growth. Two representative strains were tested per species: *A. baumannii* (YCRAb552, YCRAb667), *K. pneumoniae* (SCH530, SCH777), *E. coli* (FORC82, JSMCR1), and *P. aeruginosa* (SMC-U9, SMC-U11). Fractional inhibitory concentration index (FICI) values were calculated from each checkerboard assay, and the minimum FICI value for each strain is indicated within the corresponding panel. Synergy, additive, and indifferent interactions were defined as FICI < 0.5, 0.5–1.0, and >1.0–4.0, respectively. For *P. aeruginosa* SMC-U9 and SMC-U11, the MIC of PA74 alone exceeded the highest tested concentration; thus, the maximum tested concentration (128 µg/mL) was used as a surrogate MIC for FICI calculation. Data are representative of three independent experiments.


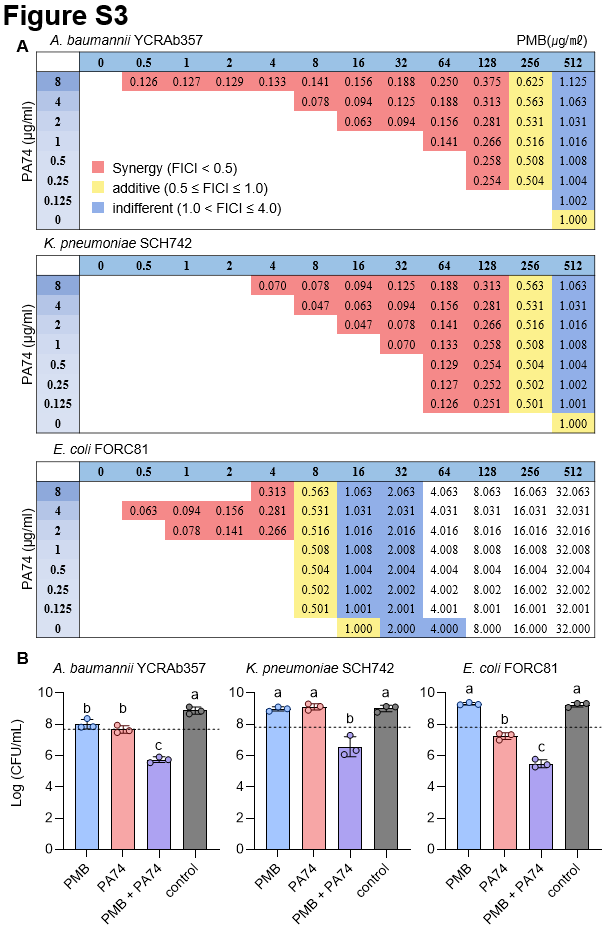


**Supplementary Figure S3.** Synergistic interaction between PA74 and PMB evaluated by checkerboard and CFU-based killing assays.

**(A)** FICI values for PA74 and PMB combinations against polymyxin-resistant Gram-negative strains. FICI values were calculated as: (MIC of PA74 in combination / MIC of PA74 alone) + (MIC of PMB in combination / MIC of PMB alone). Color coding indicates interaction type: red, synergy (FICI < 0.5); yellow, additive (0.5 ≤ FICI ≤ 1.0); blue, indifferent (1.0 < FICI ≤ 4.0). *P. aeruginosa* SMC-U10 was excluded because the FICI value did not meet the criteria for synergy (FICI < 0.5). For *K. pneumoniae* SCH742 and *E. coli* FORC81, the MIC of PA74 alone exceeded the highest tested concentration; thus, the maximum tested concentration was used for FICI estimation. Blank cells indicate concentration combinations that did not result in growth inhibition and were therefore not included in FICI calculations.

**(B)** Bacterial viability following treatment with PMB, PA74, or their combination. *A. baumannii* YCRAb357, *K. pneumoniae* SCH742, and *E. coli* FORC81 were treated with PMB and PA74 at the indicated concentrations (YCRAb357 and SCH742: 16 μg/mL PMB, 2 μg/mL PA74; FORC81: 2 μg/mL PMB, 2 μg/mL PA74) and incubated for 20 h prior to CFU enumeration. Bacterial counts are expressed as log(CFU/mL). The dashed line indicates the initial bacterial density at 0 h. Statistical significance was determined by one-way ANOVA followed by Tukey's multiple comparisons test; different letters above bars indicate statistically significant differences (*P* < 0.05).

**
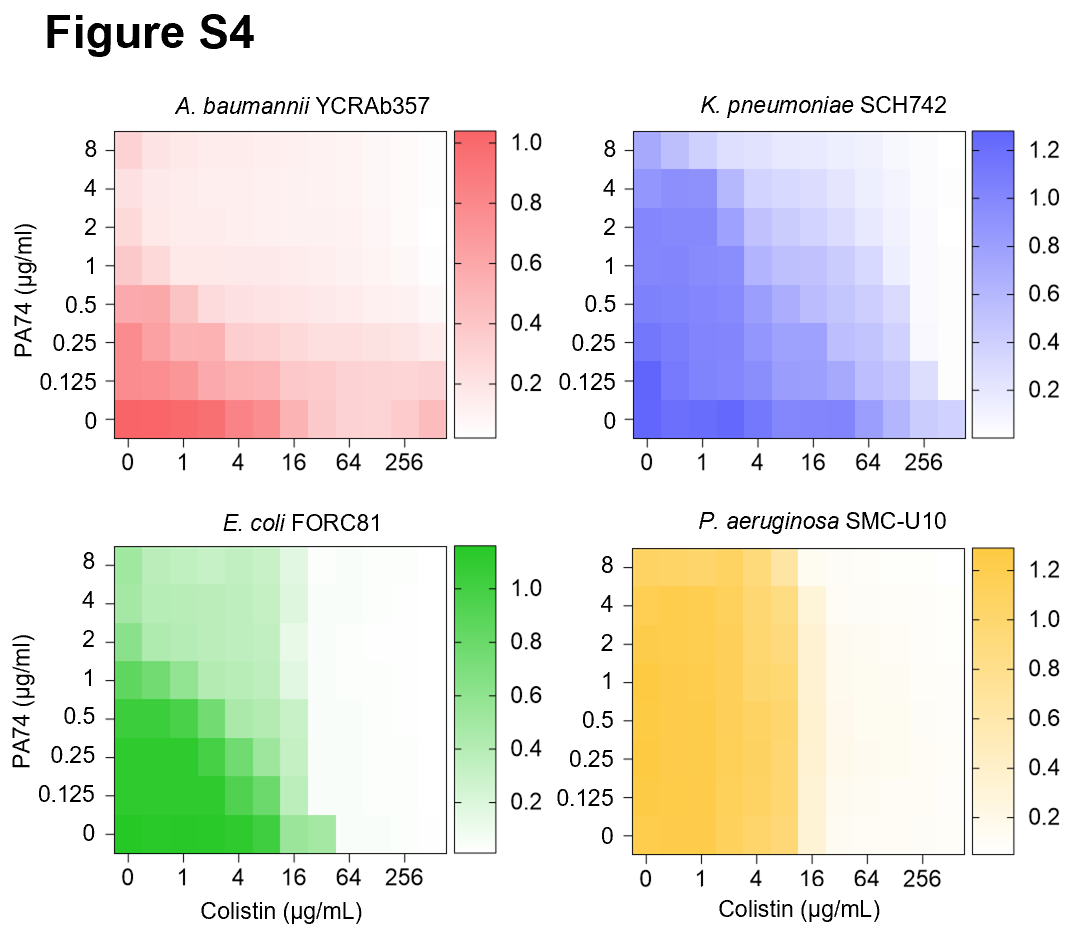
**

**Supplementary Figure S4.** Checkerboard assays evaluating the combined effects of PA74 and colistin against polymyxin-resistant Gram-negative strains. Checkerboard assays evaluating the combined effects of PA74 and colistin against representative Gram-negative clinical isolates: *A. baumannii* YCRAb357, *K. pneumoniae* SCH742, *E. coli* FORC81, and *P. aeruginosa* SMC-U10. All strains were classified as polymyxin-resistant clinical isolates. Heatmaps display normalized bacterial growth (OD_600_) across concentration matrices of PA74 (y-axis) and colistin (x-axis), with color intensity reflecting relative growth compared to the untreated control, where higher values indicate greater bacterial growth. Data are representative of three independent experiments.

**
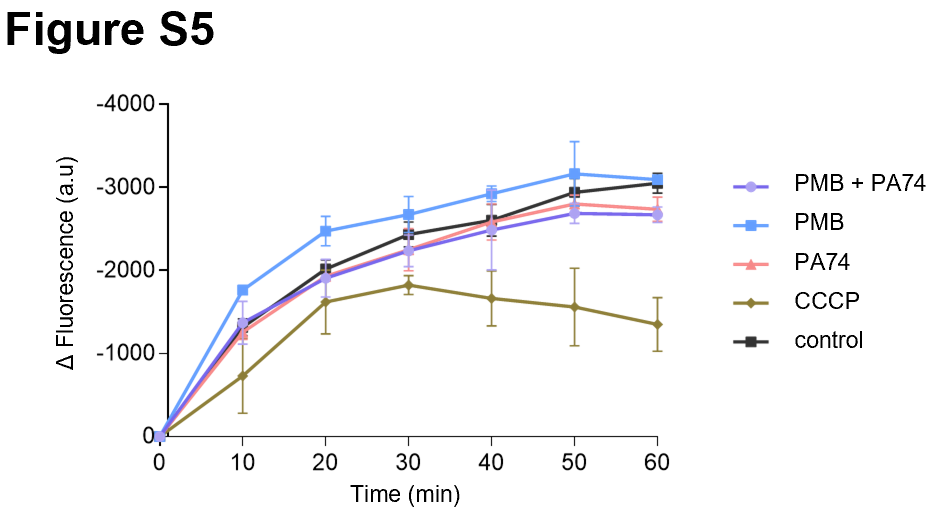
**

**Supplementary Figure S5.** Ethidium bromide (EtBr) accumulation-based efflux assay in *A. baumannii* YCRAb357.
Cells were loaded with EtBr (1 μg/mL) and treated with PMB (16 μg/mL), PA74 (2 μg/mL), PMB + PA74, CCCP (1 μg/mL), or left untreated. Fluorescence was measured every 5 min for 60 min using a microplate reader (Spark™ 10M, Tecan) at excitation/emission wavelengths of 360/460 nm. Data are expressed as Δfluorescence (Fₜ − F₀), where F₀ represents baseline fluorescence.

**
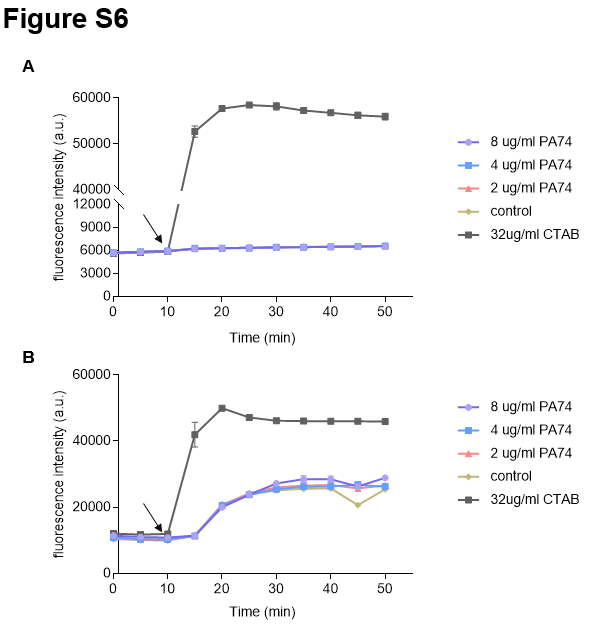
**

**Supplementary Figure S6. Membrane integrity and depolarization assays of PA74-treated *A. baumannii* YCRAb357.**

(A) Membrane integrity was assessed using propidium iodide (PI). Bacterial cells were treated with PA74 at the indicated concentrations (8, 4, and 2 μg/mL), CTAB (32 μg/mL), or DMSO (control), and fluorescence was monitored using a microplate reader (Spark™ 10M, Tecan) at excitation/emission wavelengths of 540/610 nm.

(B) Membrane depolarization was evaluated using DiSC₃(5). Bacterial cells were treated with PA74 at the indicated concentrations (8, 4, and 2 μg/mL), CTAB (32 μg/mL), or DMSO (control), and fluorescence was monitored at excitation/emission wavelengths of 635/670 nm.

In both assays, fluorescence was recorded every 5 min, and the graphs represent measurements from 10 min prior to drug treatment to 40 min after treatment. Arrows indicate the time point at which the compounds were added.


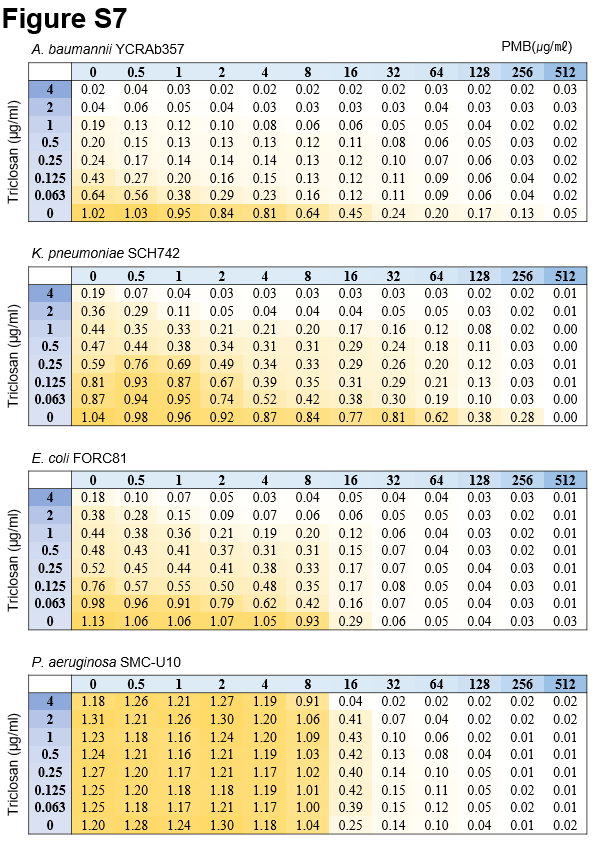


**Supplementary Figure S7.** Checkerboard analysis of PMB and triclosan combination against polymyxin-resistant Gram-negative strains. Checkerboard assays were performed to evaluate the combinatorial antibacterial activity of PMB and triclosan (TCS) against representative polymyxin-resistant Gram-negative clinical isolates: *A. baumannii* YCRAb357, *K. pneumoniae* SCH742, *E. coli* FORC81, and *P. aeruginosa* SMC-U10. Normalized bacterial growth (OD_600_) is presented across a matrix of TCS (4–0.063 µg/mL) and PMB (0.5–512 µg/mL) concentrations.


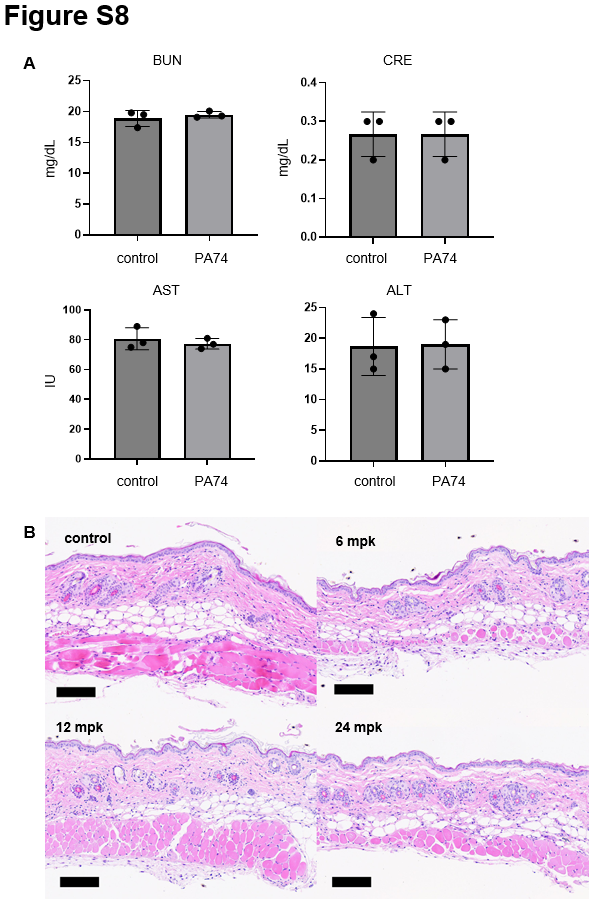


**Supplementary Figure S8. In vivo toxicity assessment of PA74.**
(A) Serum biochemical analysis following acute systemic administration of PA74. Female C57BL/6 mice were administered a single intraperitoneal dose of PA74 (200 mg/kg) or vehicle control (n = 3 per group). At 24 h post-administration, blood samples were collected, and serum levels of blood urea nitrogen (BUN), creatinine (CRE), aspartate aminotransferase (AST), and alanine aminotransferase (ALT) were measured using an automated biochemical analyzer.

**(B)** Histopathological analysis of local skin toxicity following intradermal administration of PA74. Mice received intradermal injections of PA74 at indicated doses (6, 12, and 24 mg/kg) or vehicle control at separate dorsal skin sites (n = 3). At 24 h post-injection, skin tissues were collected and histologically evaluated by hematoxylin and eosin (H&E) staining. Scale bars, 100 μm.
