# Supplementary material for: An aryloxyphenol potentiates polymyxin against multidrug-resistant Acinetobacter baumannii
Source: Front Cell Infect Microbiol. 2026 Jun 4;16:1814325. doi: 10.3389/fcimb.2026.1814325 (PMC13275670; doi:10.3389/fcimb.2026.1814325)
Supplement: Supplementary file 2 [file DataSheet2.docx]

Supplementary Material

# Supplementary Tables

**Supplementary Table S1. Bacterial strains and plasmids in this study**

| **Gram negative strains** | | **Sources** |
| --- | --- | --- |
| *Escherichia coli* BW25113 | | Laboratory stock |
| *Escherichia coli* BW25113-*mcr-1*; PMB^R^ | | This study |
| *Escherichia coli* MG1655 | | Laboratory stock |
| *Escherichia coli* SQ110 Δ*tolC* | | (Orelle et al., 2013) |
| *Escherichia coli* SQ110 *lptD*Δ330–352 | |  |
| **Clinical isolates** | | **Sources** |
| *Acinetobacter baumannii* YCRAb357; PMB^R^ | | Yonsei Severance Hospital,  Seoul, S. Korea |
| *Acinetobacter baumannii* YCRAb552; PMB^R^ | |  |
| *Acinetobacter baumannii* YCRAb667; PMB^R^ | |  |
| *Klebsiella pneumoniae* SCH530; PMB^R^ | | Samsung Changwon Hospital,  Seoul, S. Korea |
| *Klebsiella pneumoniae* SCH742; PMB^R^ | |  |
| *Klebsiella pneumoniae* SCH777; PMB^R^ | |  |
| *Pseudomonas aeruginosa* SMC-U9; PMB^R^ | |  |
| *Pseudomonas aeruginosa* SMC-U10; PMB^R^ | |  |
| *Pseudomonas aeruginosa* SMC-U11; PMB^R^ | |  |
| *Escherichia coli* FORC81; PMB^R^; *mcr-1* | | (Kim et al., 2019) |
| *Escherichia coli* FORC82; PMB^R^; *mcr-1* | |  |
| *Escherichia coli* JSMCR1; PMB^R^ | |  |
| **Plasmids** | **Relevant characteristics** | **Sources** |
| pMo130-TelR | Allelic exchange vector; TelR | Addgene #50799 (Amin et al., 2013) |
| pCA24N | IPTG-inducible promoter P_T5-lac_; Cm^R^ | (Kitagawa et al., 2005) |
| pCA24N::*fabI* | Encodes *fabI* gene in pCA24N;  GFP deletion strain |  |
| pCA24N::*fabI*(G93V) | *fabI*(G93V) in pCA24N | This study |
| pCA24N::*fabI*(F203L) | Encodes *fabI*(F203L) in pCA24N | This study |

**Supplementary Table S2. Primers used in this study**

| **Primers** | **Sequences (5’ to 3’)** | **Reference** |
| --- | --- | --- |
| mcr-1-con-F | CATCTCAGCAAGTAGGCGTT | This study |
| mcr-1-con-R | GAATTGCCGCAATTATCCCAC |  |
| FabI_G93V_F | CACTCTATTGTTTTTGCACC |  |
| FabI_G93V_R | GGTGCAAAAACAATAGAGTG |  |
| FabI_F203L_F | TCAAAGACTTGCGCAAAATG |  |
| FabI_F203L_R | CATTTTGCGCAAGTCTTTGA |  |
| pCA24N F-CA | CATTAAAGAGGAGAAATTAACTATGAGAGG |  |
| pCA24N R2 | AGTCACGATGAATTCCCCTAGCTTG |  |
| pCA24N-XhoⅠ | CGTCTTCACCTCGAGAAATC |  |
| pCA24N-NotⅠ | TCGACCCTTAGCGGCCGC |  |

**Supplementary Table S3.** Antimicrobial susceptibility profiles (MICs) of bacterial strains used in this study.

| **Species** | **Strain** | **PMB** | **TET** | **CHL** | **AMP** | **GEN** | **KAN** | **NOR** |
| --- | --- | --- | --- | --- | --- | --- | --- | --- |
|  |  | **(μg/mL)** | | | | | | |
| *Acinetobacter baumannii* | YCRAb357 | 512 | > 512 | 256 | > 512 | > 512 | > 512 | > 512 |
|  | YCRAb667 | 128 | 8 | 128 | > 512 | > 512 | 512 | 512 |

ᵃPMB, polymyxin B; TET, tetracycline; CHL, chloramphenicol; AMP, ampicillin; GEN, gentamicin; KAN, kanamycin; NOR, norfloxacin.

ᵇMIC values (μg/mL) were determined by broth microdilution and defined as the lowest concentration resulting in ≤10% of the growth of the untreated control.

**References**

Amin, I.M., Richmond, G.E., Sen, P., Koh, T.H., Piddock, L.J., and Chua, K.L. (2013). A method for generating marker-less gene deletions in multidrug-resistant Acinetobacter baumannii. *BMC Microbiol* 13**,** 158.

Kim, J., Hwang, B.K., Choi, H., Wang, Y., Choi, S.H., Ryu, S., and Jeon, B. (2019). Characterization of mcr-1-Harboring Plasmids from Pan Drug-Resistant Escherichia coli Strains Isolated from Retail Raw Chicken in South Korea. *Microorganisms* 7.

Kitagawa, M., Ara, T., Arifuzzaman, M., Ioka-Nakamichi, T., Inamoto, E., Toyonaga, H., and Mori, H. (2005). Complete set of ORF clones of Escherichia coli ASKA library (a complete set of E. coli K-12 ORF archive): unique resources for biological research. *DNA Res* 12**,** 291-299.

Orelle, C., Carlson, S., Kaushal, B., Almutairi, M.M., Liu, H., Ochabowicz, A., Quan, S., Pham, V.C., Squires, C.L., Murphy, B.T., and Mankin, A.S. (2013). Tools for characterizing bacterial protein synthesis inhibitors. *Antimicrob Agents Chemother* 57**,** 5994-6004.
